# Controlled release of antimicrobial peptides from nanocellulose wound dressings for treatment of wound infections

**DOI:** 10.1016/j.mtbio.2025.101756

**Published:** 2025-04-10

**Authors:** Elisa Zattarin, Zeljana Sotra, Emanuel Wiman, Yagmur Bas, Jonathan Rakar, Linn Berglund, Annika Starkenberg, Emma M. Björk, Hazem Khalaf, Kristiina Oksman, Torbjörn Bengtsson, Johan P.E. Junker, Daniel Aili

**Affiliations:** a Laboratory of Molecular Materials, Division of Biophysics and Bioengineering, Department of Physics, Chemistry and Biology, Linköping University, SE-581 83, Linköping, Sweden; b Center for Disaster Medicine and Traumatology, Department of Biomedical and Clinical Sciences, Linköping University, SE-581 85, Linköping, Sweden; c Department of Microbiology, Immunology and Reproductive Science, School of Medical Sciences, Örebro University, SE-70362, Örebro, Sweden; d Division of Materials Science, Department of Engineering Sciences and Mathematics, Luleå University of Technology, SE-971 87, Luleå, Sweden; e Division of Nanostructured Materials, Department of Physics, Chemistry and Biology (IFM), Linköping University, SE-58183, Linköping, Sweden

**Keywords:** Wound dressing, Wound infection, Nanocellulose, Antimicrobial peptides, Bacteriocin, PLNC8

## Abstract

Wounds are highly prone to infection, which can delay healing and lead to severe complications such as gangrene and sepsis. Non-healing wounds significantly impact patients' physical and mental well-being and place a substantial financial burden on healthcare systems. Timely and effective treatment of wound infections is critical, but the rise of antibiotic-resistant pathogens complicates this process. In this study, we investigate a potent protease resistant antimicrobial peptide (AMP), PLNC8 αβ, for the treatment of wound infections and present a strategy for localized AMP delivery using functionalized advanced nanocellulose (NC) wound dressings. Two types of NC dressings were explored: bacterial cellulose (BC) and TEMPO-oxidized nanocellulose derived from wood powder (TC). In a porcine wound infection model, PLNC8 αβ exhibited high antimicrobial activity, successfully eradicating the infection while promoting wound re-epithelialization. To achieve controlled release of PLNC8 αβ from the NC dressings, the peptides were either physisorbed directly onto the nanofibrils or encapsulated within mesoporous silica nanoparticles (MSNs) that were incorporated into the dressings. The PLNC8 αβ functionalized dressings demonstrated low cytotoxicity toward human primary fibroblasts and keratinocytes. Both BC and TC dressings showed efficient contact inhibition of bacteria but were less effective in inhibiting bacteria in suspension. In contrast, MSN-functionalized dressings, displayed significantly enhanced peptide-loading and sustained release capacities, resulting in improved antimicrobial efficacy. These findings highlight the potential of PLNC8 αβ and PLNC8 αβ-functionalized nanocellulose wound dressings for the treatment of infected wounds, offering an effective alternative to conventional antibiotic therapies.

## Graphical abstract

**Figure:**
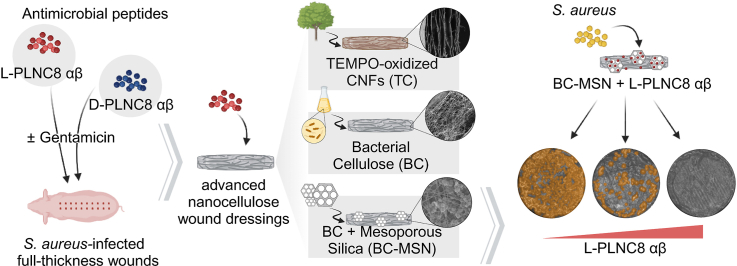

## 1 Introduction

Wound infections remain a significant clinical challenge, contributing to delayed healing and increased healthcare costs, and aggravating the global burden of antimicrobial resistance. The impact of wound infections on the quality-of-life for patients can be tremendous [1], resulting in persistent pain, wound chronification, and increased morbidity and mortality [2]. In addition, wound infections pose an enormous burden on the healthcare system. Infections occur in over 20 % of burn wounds [3], over 50 % of diabetic chronic ulcers [4] and in about 3.6 % of all surgical wounds (surgical site infections, SSI) [5], thus consuming significant health care resources [6]. Conventional treatment of infected wounds include surgical or enzymatic wound debridement and systemic administration of antibiotics [3,7]. Systemic antibiotic therapy can be effective in well-perfused wounds, but in wounds with poor vascularization, such as diabetic foot ulcers, pressure ulcers, and ischemic arterial wounds, the delivery of systemic antibiotics can be impaired [7]. Local delivery can facilitate administration of high concentrations of antibiotics in the wound while avoiding adverse systemic side effects [1,7,8]. However, the formation of biofilms by wound pathogens, such as *S. aureus* and *P. aeruginosa,* can drastically reduce the efficacy of antibiotics irrespectively of the route of administration [3,8,9]. Treatment of wound infections is further complicated by the rapid development of antibiotic resistance in the most common wound pathogens, the so-called ESKAPE pathogens (*Enterococcus faecium, Staphylococcus aureus, Klebsiella pneumoniae, Acinetobacter baumannii, Pseudomonas aeruginosa,* and *Enterobacter species*) [7,10]. Multidrug resistance has become a severe global threat that results in increased hospitalization and mortality [3,10].

Antimicrobial peptides (AMPs) have been extensively investigated as alternatives to antibiotics for treatment of wound infections [11]. AMPs are typically short cationic and amphipathic peptides that are less prone to resistance development compared to conventional antibiotics [9]. Unfortunately, AMPs display short *in vivo* half-life and can induce adverse effects in humans when administered systemically [12]. Topical AMP delivery can potentially circumvent these issues [13]. Conjugation of AMPs directly to a wound dressings can facilitate localized antimicrobial effect while minimizing the cytotoxic side effects [[14], [15], [16], [17]]. However, the covalent tethering of the AMPs can compromise their interaction with target microbes [18], induce structural changes in the peptides that reduce their efficacy [19,20], and prevent AMP penetration into the wound tissue [8]. Moreover, the chemistries required for AMP immobilization could impact the properties of the dressings and reduce their biocompatibility.

Nanocellulose-based wound dressings, particularly those derived from bacterial cellulose (BC), represent a significant advancement in wound care due to their unique structural, mechanical, and biofunctional properties [21]. BC is comprised of a nanofibrillar cellulose network, providing high surface area, gas permeability, and high fluid retention capacity, which promotes moist wound healing and stimulates tissue regeneration [21]. BC is non-toxic, and non-immunogenic, reducing the risk of inflammatory responses. The high flexibility, tensile strength and mechanical stability of BC allow for conformability to irregular wound surfaces. Commercially available nanocellulose wound dressings based on BC (Epiprotect™, Epicite hydro, XCell®, Vermac®, Nanoderm™, Dermafill®), and wood cellulose based dressings (FibDex®), are widely used in the clinic [22]. Since nanocellulose is non-degradable by human enzymes, they will remain intact and can sit on the wound for extended periods of time. However, nanocellulose is not inherently antimicrobial, and bacteria can thrive in the protected moist wound microenvironment under the dressings [23].

The functionalization of nanocellulose wound dressings with antimicrobials is complicated by the inert nature of the nanofibrillar materials. To avoid the need for harsh chemical treatment required to introduce functional groups in bacterial nanocellulose (BC) dressings, Jančič et al. [24] included carboxymethyl cellulose in the culture medium of the BC producing bacteria to facilitate the covalent immobilization the AMP nisin [24]. AMPs have also been covalently conjugated to carbohydrate binding peptides and other nanocellulose binding moieties, facilitating the loading of AMPs in the dressings [[25], [26], [27]]. Although these materials demonstrated potent contact-killing properties *in vitro*, their efficacy *in vivo* has not yet been explored [[25], [26], [27]]. Moreover, the strong interactions between the AMPs and the dressing materials can reduce AMP efficacy. Less-strongly bound AMPs tend to enable more efficient AMP delivery into the wound [18], which has been explored by incorporating AMPs during electrospinning [[28], [29], [30], [31], [32]], by immersion loading [33], by loading of AMPs in embedded nanoparticles [34], and by AMP physisorption to the dressing components [16,35]. However, achieving sufficiently high local concentrations of AMPs to treat a wound infection and tuning the release kinetics to maintain the antibacterial effect over a sufficiently long period of time remain very challenging. Moreover, both bacterial proteases and overexpression of proteases by the host because of the inflammatory response make AMPs highly susceptible to proteolytic degradation in infected wounds [8,36]. The life-time of AMPs, such as LL-37, in infected wounds are consequently very short [9].

**Fig. 1:**
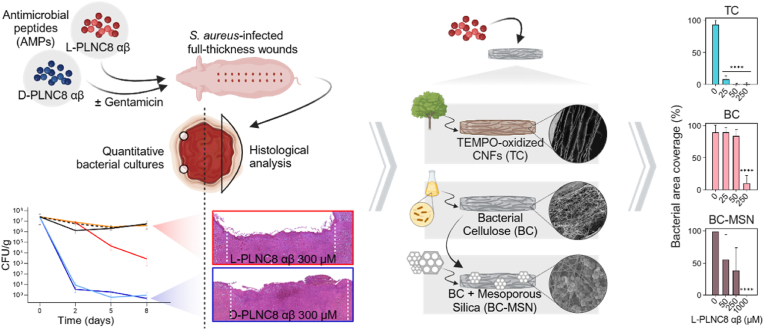
Treatment of *S. aureus*-infected full thickness wounds with antimicrobial peptides PLNC8 αβ, quantitative bacterial cultures and histological analysis at endpoint (left). Antimicrobial wound dressings were obtained by adsorbing the antimicrobial peptide PLNC8 αβ in TEMPO-oxidized nanocellulose (TC) and bacterial cellulose (BC) wound dressings. To further increase the capabilities to release large quantities of PLNC8 αβ form BC wound dressings, BC was functionalized with mesoporous silica nanoparticles (MSNs) (middle). *S. aureus* coverage (%) on surface of loaded dressings after direct inoculation and 24 h incubation of PLNC8 αβ-loaded dressings (right).

Here we show the efficacy of the protease stable two-peptide bacteriocin PLNC8 αβ using an infected porcine wound model for treatment of wound infections. Moreover, we demonstrate a novel controlled release system based on nanocellulose wound dressings for localized delivery of PLNC8 αβ at concentrations that are sufficiently high to eliminate wound infections (Fig. 1). PLNC8 αβ efficiently triggers disruption of bacterial membranes and is highly effective against both gram-positive bacteria (*S. aureus* and *Staphylococcus epidermidis*) and gram-negative bacteria (*Porphyromonas gingivalis*) *in vitro* [[37], [38], [39]]. Importantly, PLNC8 αβ is functional and highly active when synthesized using D-amino acids [38]. The D-isomer of PLNC8 αβ is inert to proteolytic degradation and thus an attractive option for treatment of infected wounds with high protease activity [38,40]. Furthermore, these peptides show limited cytotoxicity towards eukaryotic cells at the concentrations required to kill bacteria [38]. The use of PLNC8 αβ in combination with conventional antibiotics also results in synergistic effects that drastically reduce the concentrations of antibiotics required to obtain bactericidal effects [38]. We show that PLNC8 αβ is well tolerated by human primary dermal fibroblasts and keratinocytes *in vitro* and could completely irradicate severe wound infections in an infected porcine wound model, while promoting wound re-epithelialization. However, due to haemolytic effects at high concentrations [38], systemic delivery of PLNC8 αβ should be avoided. We therefore developed a strategy for release of PLNC8 αβ from advanced nanocellulose wound dressings to optimize the localized delivery in wounds.

Here, both a commercial BC wound dressing (Epiprotect™) and a designed BC-mimetic wood derived nanocellulose dressing (TC) were explored as carriers for PLNC8 αβ [[41], [42], [43], [44], [45], [46], [47], [48], [49]]. The TC dressings were prepared by direct TEMPO oxidation of wood powder followed by templating by vacuum filtration [49], offering a cost effective alternative to BC. Both dressings show excellent conformability and water retention capacity, forming a tight interaction with the wound while maintaining a moist wound environment, which can reduce pain and stimulate healing [3,4,50]. The sub-mm thickness and optical transparency of the dressings further allows for visual inspection of the wound bed without removal of the dressings. Transparency further facilitates the integration of optical sensors for wound monitoring [51]. In contrast to most other dressings, that require frequent dressings changes, nanocellulose dressings can remain on the wound for weeks, which can drastically reduce wound care costs and increase quality-of-life for patients [[42], [43], [44]]. The sub-micron porosity of the fibrillar nanocellulose network in both BC and TC can prevent bacteria penetration and reduce the risk for external contamination of the wound after dressing application. However, bacteria already present in the wound can thrive in the moist and protected environment underneath the dressings, highlighting the need for localized delivery of antimicrobials. The PLNC8 αβ functionalized nanocellulose wound dressings developed in this study combine excellent wound dressing properties with delivery of very potent antimicrobial peptides, which can further expand the versatility of BC and TC wound dressings and offer a novel and efficient strategy for treatment of infected wounds.

## 2 Results and discussion

### 2.1 Evaluation of PLNC8 αβ cytotoxicity

**Fig. 2:**
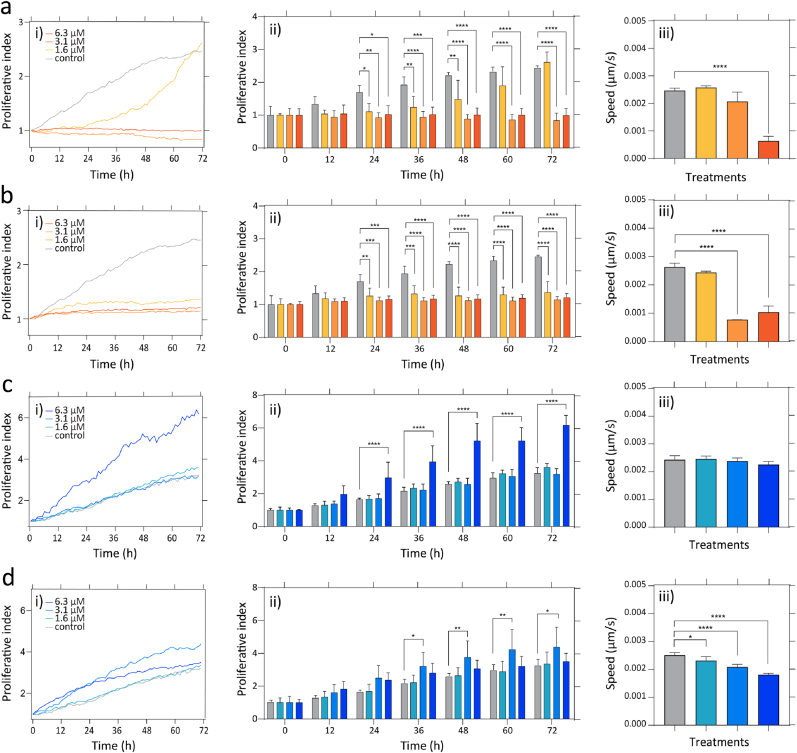
Proliferation and cell migration speed over time following exposure to PLNC8 αβ. Keratinocytes exposed to a) L-PLNC8 αβ, b) D-PLNC8 αβ, and fibroblasts exposed to c) L-PLNC8 αβ and d) D-PLNC8 αβ. Results displayed as mean and standard deviation. Statistical analysis was carried on using a two-way ANOVA complemented with Dunnett's multiple comparison test (∗P < 0.1, ∗∗P < 0.01, ∗∗∗P < 0.001, ∗∗∗∗P < 0.0001, n.s. whereas not indicated).

The antimicrobial peptide (AMP) PLNC8 αβ has demonstrated high antimicrobial activity *in vitro* against several types of bacteria, including the common wound pathogen *S. aureus* [52]. However, *in vivo*, AMPs are susceptible to rapid proteolytic degradation by both bacterial and host proteases [13,38]. To explore the effect of proteolytic degradation on the antimicrobial activity *in vivo*, we synthesized PLNC8 αβ using D-amino acids and compared the effect to peptide synthesized using L-amino acids. We have previously demonstrated that the D-enantiomer of PLNC8 αβ can resist proteolytic degradation while retaining its antimicrobial activity *in vitro* [38]. Prior to evaluation *in vivo*, a toxicological risk assessment of the peptides was performed on human primary keratinocytes and primary dermal fibroblasts. The cells were exposed for 72 h to the L- and D-enantiomers of PLNC8 αβ in a concentration range above the minimal bactericidal concentration (MBC) of the peptides, to assess the dose-response effect. Proliferation and cell migration speed were assessed over time using ptychographic imaging. Both enantiomers of the peptides induced a significant reduction in keratinocyte proliferation after 24 h compared to the control (Fig. 2a ii) and Fig. 2b ii)). However, the lowest concentration of the L-form (1.8 μM) was tolerated with minimal effects even after 60 h (2.33 ± 0.13 for control and 1.90 ± 0.57 for L-PLNC8 αβ, expressed as mean and SD factor, respectively, *p* < 0.05; Fig. 2a ii). The highest concentration of the L-form tested (6.3 μM) caused a significant increase in proliferation of the fibroblasts after a 24 h exposure (1.64 ± 0.08 for control and 2.97 ± 0.95 for L-PLNC8 αβ, expressed as mean and SD factor, *p* < 0.05; Fig. 2c ii), whereas 3.1 μM of the D-form caused an increase in proliferation after 36 h (2.16 ± 0.23 for control and 3.21 ± 0.83 for D-PLNC8 αβ, expressed as mean and SD factor, *p* < 0.05; Fig. 2d ii). Higher concentrations of the peptides were tolerated in terms of proliferation with respect to the control. Significant effects on average cell migration speed were observed for keratinocytes treated with 6.3 μM L-PLNC8 αβ, 3.1 μM and 6.3 μM D-PLNC8 αβ and for fibroblasts treated with 1.8 μM, 3.1 μM and 6.3 μM D-PLNC8 αβ. No migratory speed reduction was observed for fibroblasts exposed to L-PLNC8 αβ. Thus, some cytotoxicity was observed, particularly on keratinocytes. These findings contradict previous observations by Bengtsson et al., [38] showing increased proliferation of both keratinocyte and fibroblast cell lines after treatment with the peptides. Here we used primary cultures, which could explain the differences. The observed differences in fibroblast viability between L- and D-PLNC8 αβ may relate to subtle variations in their interaction with cellular membranes, degradation rate, or internalization mechanisms, which warrant further mechanistic investigation in future studies. Based on the absence of cytotoxic effects in the relevant concentration range, both enantiomers of PLNC8 αβ were further evaluated *in vivo* to assess efficacy and effects on the wound healing process.

### 2.2 Efficacy of PLNC8 αβ *in vivo*

**Fig. 3:**
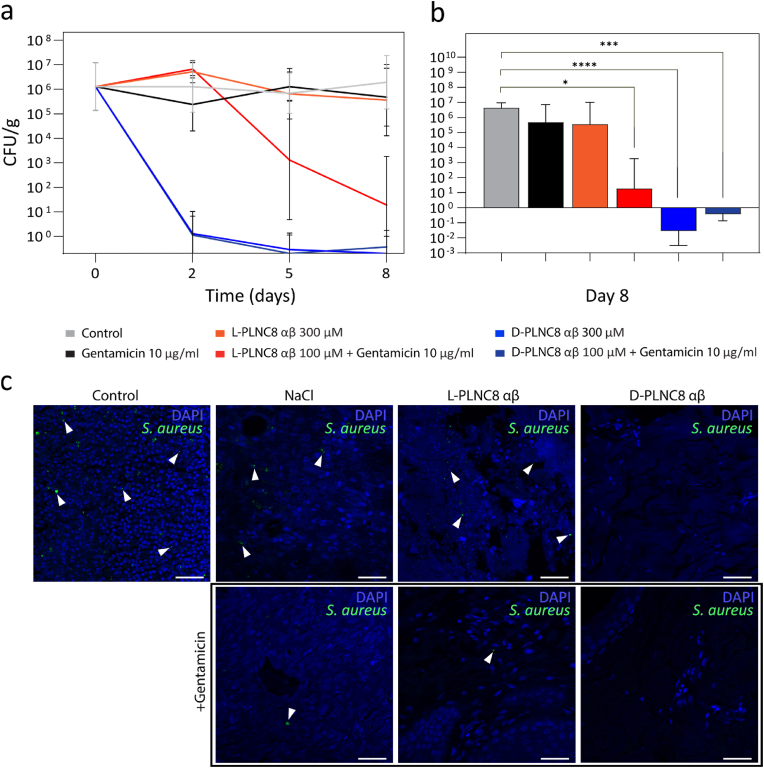
*In vivo* evaluation of PLNC8 αβ using a porcine infected wound model. a) Progress of tissue infection expressed as number of colony forming units per gram of tissue (CFU/g) at day 0, 2, 5, and 8 post infection, obtained by quantitative cultures of tissue biopsies. b) Tissue infection expressed as number of colony forming units per gram of tissue (CFU/g) at day 8 post infection. Treatment with PLNC8 αβ was initiated at Day 2. c) Immunofluorescence staining of *S. aureus* colonies (green, indicated by arrows) in tissue biopsies on day 8. Cell nuclei were counterstained with DAPI (blue). Scale bar: 100 μm. (For interpretation of the references to color in this figure legend, the reader is referred to the Web version of this article.)

An infected porcine wound model was used to evaluate the antimicrobial activity of PLNC8 αβ *in vivo* and the impact of the peptides on wound healing. The porcine wound model represents the highest fidelity preclinical model in terms of translatability, due to anatomical and histological similarities to human skin and wound healing process [53,54]. In addition, both human and porcine wounds heal through re-epithelialization, display equivalent immune cell responses, and share similarities in epidermal and dermal thickness, hair follicles and dermal metabolism [53,54]. Circular wounds (1 cm diameter) were created using biopsy punches and inoculated with 10^7^ CFU *S. aureus* two days prior to treatment. Treatment was applied topically and consisted of 300 μM L/D-PLNC8 αβ, 10 μg/ml gentamicin, saline solution as a control, as well as a combination of gentamicin (10 μg/ml) with L/D-PLNC8 αβ (100 μM). Quantitative cultures on selective agar were performed on biopsies obtained from timepoints 0, 2, 5 and 8 days. The cultures revealed that treatment solely with gentamicin or L- PLNC8 αβ did not significantly reduce infection and was comparable to the saline control (4.8 × 10^5^ ± 15.01, 3.6 × 10^5^ ± 28.16, and 4.3 × 10^6^ ± 2.17 CFU/mg, expressed as geometric mean and geometric SD factor, respectively, *p* > 0.999; Fig. 3a). However, the combination of L-PLNC8 αβ and gentamicin resulted in a reduction in the infection of several orders of magnitude at day 8 post inoculation (1.9 × 10^1^ ± 96.25 for the combined treatment, expressed as geometric mean and geometric SD factor, *p* < 0.05; Fig. 3b), confirming the previously observed synergistic effects when combining PLNC8 αβ with antibiotics [38]. Interestingly, D-PLCN8 αβ completely eradicated the infection resulting in bacterial counts below 10^1^ CFU/g both alone (2.79 × 10^−2^ ± 8.88, expressed as geometric mean and geometric SD factor, *p* < 0.0001; Fig. 3b) and in combination with gentamicin (3.67 × 10^−1^ ± 2.72, expressed as geometric mean and geometric SD factor, p < 0.005; Fig. 3b). These observations thus clearly show that D-PLNC8 αβ was highly effective in reducing wound bioburden. This was further confirmed by immunofluorescence staining showing absence of bacteria in the wounds treated with D-PLCN8 αβ (Fig. 3c).

In contrast, immunofluorescence staining of wound treated with L-PLNC8 αβ did not show any significant decrease in the level of infection and bacteria was still present in the wound after treatment. The difference in efficacy between the enantiomers is likely due to the proteolytic stability of the D-peptide, which aligns with previous *in vitro* findings by Bengtsson et al. [38] The L-peptide is thus likely degraded during the time course of the treatment whereas the D-peptide remains intact and functional. The synergistic effect seen when combining L-PLNC8 αβ with gentamicin, which resulted in a bioburden level comparable to normal flora in terms of CFU/g, can be the result of multiple different factors [55], such as the AMP-mediated disruption of membrane integrity that could facilitate entry of antibiotics into the cytoplasm [55,56], or by disruptions of the biofilm matrix that could increase the antibiotic susceptibility of biofilm-protected bacteria. Irrespectively, the possibilities to reduce infection by combining low doses of AMPs and antibiotics could reduce toxicity, prevent microbial resistance development, and enhance clinical outcomes [56].

**Fig. 4:**
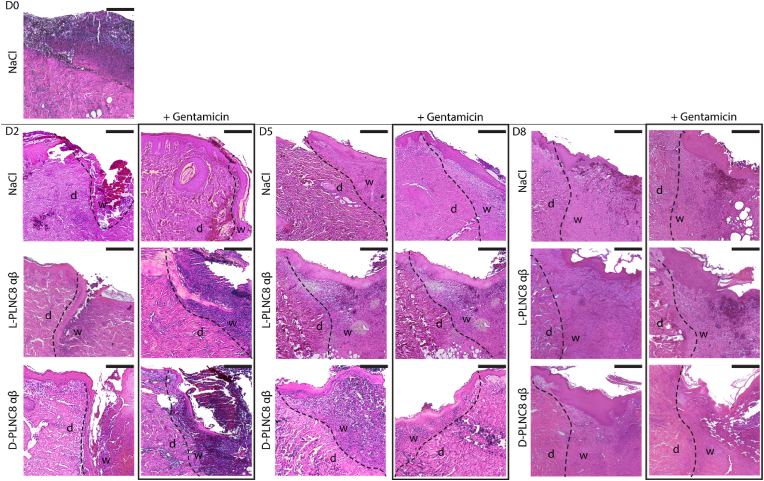
Histological evaluation of PLNC8 αβ on wound healing. Biopsies of partial-thickness wounds from days 0, 2, 5 and 8 were stained with Hematoxylin-Eosin (HE). The dashed lines delineate wound borders. Left side of the lines shows healthy dermis (d), whereas the right side shows the wound area and granulation tissue (w). Scale bar: 500 μm.

A histological evaluation of Hematoxylin-Eosin (HE) stained biopsies of partial-thickness wounds from days 0, 2, 5 and 8 post-infection was performed to evaluate the impact of PLNC8 αβ on the healing process. Re-epithelialization with clear neoepidermis and an underlying granulation tissue formation was observed in the HE stained histological images at the end of treatment in all groups (Fig. 4 and Fig. S1a). Treatment with gentamicin, L-PLNC8 αβ, or a combination thereof significantly increased re-epithelialization of the wounds, with the exception of D-PLNC8 αβ alone, which did not impact healing (Fig. S1b). A slightly hyperproliferative epidermis was seen in some of the samples. Hyperproliferation may be associated with increased risk of hypertrophic scarring and keloid formation [57,58], however, it is typically only observed in the first weeks of healing [59]. Furthermore, the transition from the inflammatory to the proliferative phase in wound healing is critical for the overall healing outcome [60]. Thus, these findings of re-epithelialization with slight hyperproliferation on day 8, might suggest successful transitioning onto the later stages of the healing phases [57,58]. Moreover, formation of neoepidermis and granulation tissue was observed at day 5. The earlier timepoints (D0 and D2) instead show a wound area covered with a blood clot or an empty space. Collectively, re-epithelialization was not hindered, but rather increased by the treatments in the tested concentrations. Hence, the slight cytotoxic effects of the peptides observed *in vitro* were not observed when administered *in vivo*. Overall, PLNC8 αβ were found to be tolerated by cells and wound tissue, and demonstrated excellent antimicrobial activity *in vivo*, with complete bacterial eradication following treatment with the D-enantiomer. Combining L-PLNC8 αβ with gentamicin reduced the bioburden of the wound tissue well below clinically relevant levels, and enhanced wound healing. Based on the demonstrated efficacy of PLNC8 αβ, we continued to explore the possibilities to release the peptides from wound dressings to develop a clinically feasible wound treatment.

### 2.3 Nanocellulose wound dressing materials

**Fig. 5:**
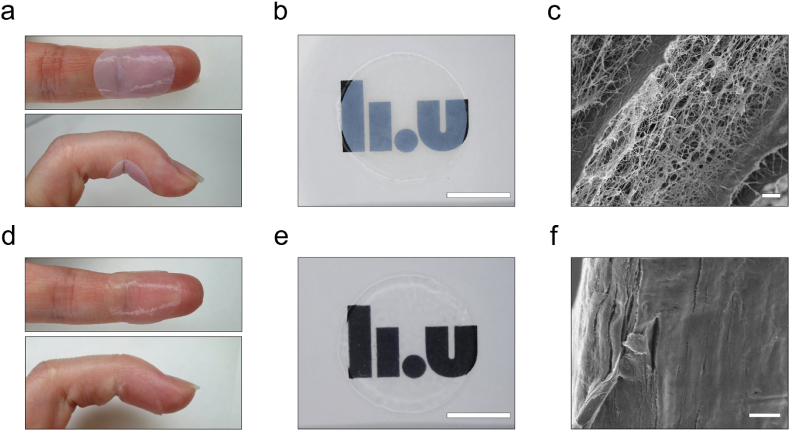
a,d) Conformability of circular (Ø 20 mm) a) BC and d) TC on intact skin. b,e) Photographs demonstrating the transparency of circular (Ø 20 mm) b) BC and e) TC dressings, scale bar: 10 mm. c,f) Scanning electron micrographs of the cross-sectional view of c) BC (scale bar: 2 μm), and f) TC (scale bar: 2 μm).

Two different types of advanced nanocellulose wound dressings were evaluated as carriers for PLNC8 αβ: a commercial wound dressing based on bacterial nanocellulose (BC) (Epiprotect™) and nanocellulose wound dressings derived from aspen wood powder (TC) [49]. The nanocellulose dry content was 7.6 g/m^2^ and 20.8 g/m^2^ for BC and TC, respectively. Both wound dressing materials were highly hydrated and had a water content of 97.2 ± 0.1 % for BC and 96.4 ± 0.7 % for TC dressings. BC and TC wound dressings had a thickness in the dry state of 11 ± 3 μm and 41 ± 7 μm, and a thickness in the wet state of 366 ± 49 μm and 399 ± 41 μm, respectively. Both materials showed a negative ζ-potential of −17.6 ± 0.1 for BC and −52 ± 2 for TC [49]. Some differences in the microarchitecture of the two dressing materials were observed. BC is produced by bacteria (*K. xylinus*) cultured under static conditions [61]. The resulting nanocellulose dressing presents a dense fibrillar network with tightly entangled fibrils with a diameter of 40–60 nm [62]. Inter- and intra-fibrillar hydrogen bonds in combination with physical entanglement contribute to the high mechanical strength and flexibility of the BC dressings (Fig. 5a and b) [61,63]. The fibrillar 3D network structure resembles the nanoscale architecture of extracellular matrix and possesses a good permeability to gases and liquids [4] (Fig. 5c).

The BC wound dressings displayed a rather large batch-to-batch variation with respect to thickness (Fig. S2a). Some differences in thickness were also seen within the same dressing as indicated by the variations in optical density (Fig. S2b), which is a consequence of the biological production method of BC. The TC dressings were produced by direct TEMPO-oxidation of hardwood powder followed by fibrillation, resulting in an aqueous CNF suspension [64]. The suspension was vacuum filtered to allow for fiber self-assembly and formation of a hydrogel. The self-assembly process was driven by the establishment of hydrogen-bonds between fibrils and resulted in the creation of a stable network of nanocellulose fibrils forming dressings that were more homogenous in thickness and density compared to BC (Fig. 5d and e). The high degree of carboxylation (0.45 ± 0.12 mmol/g) [49] also resulted in materials with a more pronounced negative charge and smaller fibrils compared to BC. The TC fibril diameter was 1.9 ± 0.9 nm [49] and the fibrils were organized in a defined layered structure (Fig. 5f). Both materials, however, show attractive properties as wound dressings, including a good conformability to the skin (Fig. 5a–d). Conformability allows the dressing to be in close contact with the wound and adapt to any movements while preventing formation of air pockets that could lead to undesired pressure/tension on the newly formed skin and the stagnation of exudates, which would lead to tissue maceration and more favorable conditions for bacterial growth [65].

### 2.4 Physisorption of PLNC8 αβ in nanocellulose wound dressings

Because of the nanofibrillar structure, BC and TC dressings have a large effective surface area, which can enable loading of high quantities of antimicrobial peptides. PLNC8 α and PLNC8 β are amphipathic and consequently tend to associate into larger micellar structures in aqueous solutions above the critical micelle concentration (CMC), both separately and when combined (Figs. S5–S8), which can influence both the BC/TC loading strategy and the peptide release kinetics. Fluorescence self-quenching of Cy3-labelled PLNC8 α peptides (Fig. S5a) and fluorescence resonance energy transfer (FRET) between Cy3-labelled PLNC8 α Cy5-labled PLNC8 β (Fig. S5b) confirmed both formation of micelles and that the two different peptides co-assembled within the same micellar structures. The hydrodynamic radius (R_H_) of the micelles was between 180 and 480 nm (Fig. S6), which is on par with the size of the pores in the nanocellulose network in the dressings. The CMC was dependent on both the ionic strength and the buffer composition (Figs. S7–S8). PLNC8 α and β demonstrated a CMC of 9 and 12.3 μM in milliQ water and 14 and 17 μM in saline, respectively. When combined in a molar ratio of 1:1, the CMC of PLNC8 αβ was 43 μM and 13 μM in milliQ water and saline solution, respectively. The CMC decreased to <0.2 μM in PBS+10 % FBS and LB broth, which indicates that the peptides will likely exist as micelles when administered under physiological conditions. Peptide-peptide charge repulsions is thus likely more pronounced in milliQ water, making the micelles more dynamic.

To load PLNC8 αβ on BC and TC dressing, the peptides were consequently dissolved in milliQ water to reduce charge shielding and promote electrostatic interactions between the positively charged peptides and the nanocellulose fibrils, to facilitate efficient penetration of the peptides into the dressings.

**Fig. 6:**
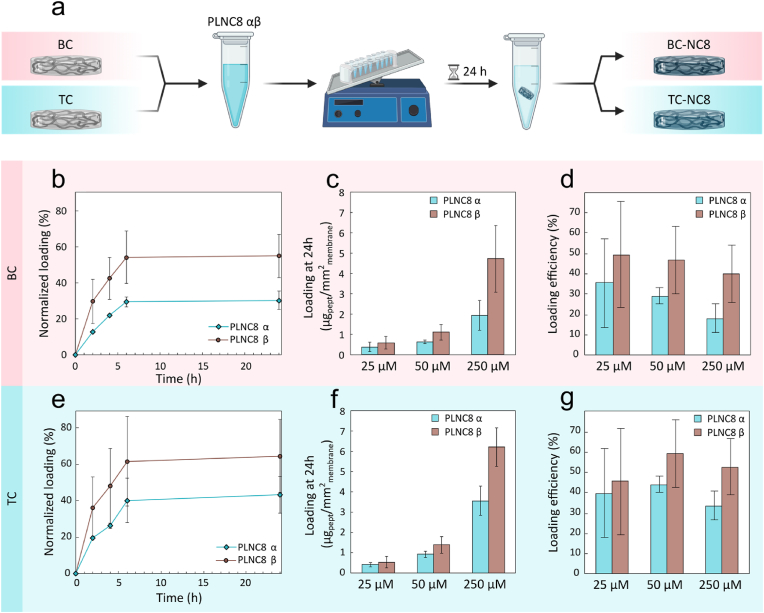
a) Schematic illustration of the peptide physisorption process in BC and TC dressings. b,e) Representative curve of the loading kinetics of peptide PLNC8 α/β (1:1, 50 μM) in b) BC and e) TC dressings. n = 2. c,f) Loaded peptide mass per unit dressing area at 24 h and d,g) loading efficiency at 24 h of incubation of peptides PLNC8 αβ (1:1 at 25, 50 and 250 μM) in c,d) BC and f,g) TC dressings.

The potential for loading the dressings with PLNC8 αβ were first explored by physisorption of the peptides directly onto the nanocellulose fibrils (Fig. 6a). The PLNC8 α and β peptides have a positive net-charge of +4.1 and +5.2 at neutral pH, respectively [38], while BC and TC have a negative ζ-potential of −17.6 ± 0.1 and −52 ± 2, respectively [49]. Hence, we hypothesized that charge-charge interactions could facilitate the adsorption of large quantities of PLNC8 αβ peptides in the dressings. To monitor the adsorption and release processes, PLNC8 α and β were labelled with sulfo-Cy3 and sulfo-Cy5, respectively. The adsorption kinetics was investigated using peptide concentrations ranging from 25 to 250 μM (PLNC8 α/β 1:1) over 24 h (Fig. 6b–e). Owing to a higher net positive charge, PLNC8 β adsorbed to a greater extent than PLNC8 α to both BC (Fig. 6c) and TC (Fig. 6f) dressings. In terms of overall peptide loading, TC dressings performed best, likely due to their higher net-negative charge. Although the amount of loaded peptide was proportional to the peptide concentration in solution, loading efficiency remained quite low in all cases, not surpassing 36 % for PLNC8 α and 50 % for PLNC8 β in BC dressings (Fig. 6d) and 40 % for PLNC8 α and 60 % for PLNC8 β in TC dressings (Fig. 6g). For the highest peptide concentration (250 μM PLNC8 αβ), we obtained a loading of 1.9 ± 0.7 μg PLNC8 α/mm^2^ and 4.7 ± 1.6 μg PLNC8/mm^2^ in the BC dressings, and 3.6 ± 0.7 μg PLNC8 α/mm^2^ and 6.2 ± 0.9 μg PLNC8 β/mm^2^ in the TC dressing.

### 2.5 Peptide release from BC and TC dressings

**Fig. 7:**
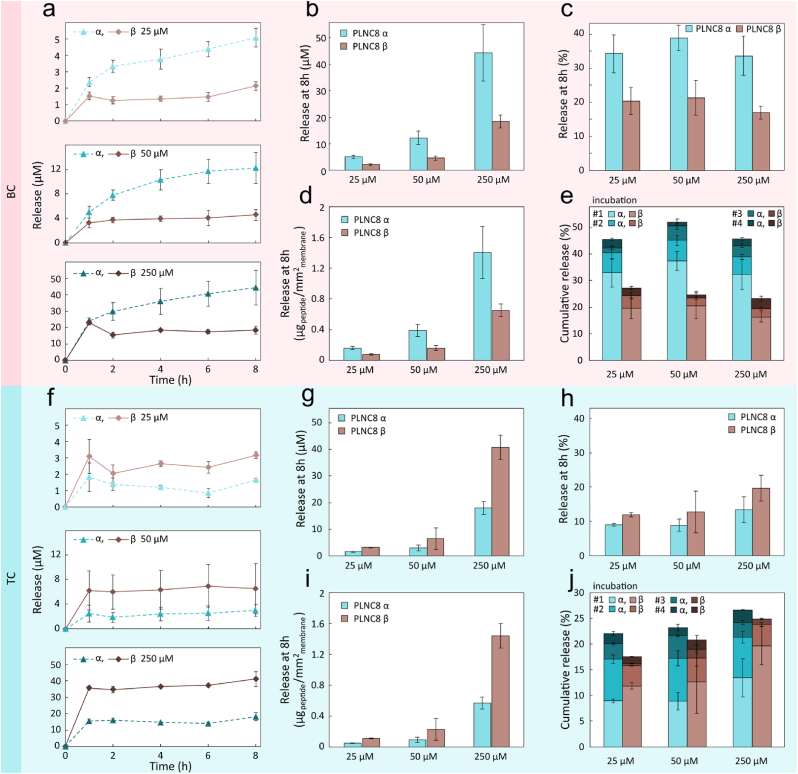
a, f) Release kinetics of PLNC8 αβ from over a period of 8 h in 250 μL PBS +10 % FBS from a) BC and f) TC dressings, after loading using 25, 50, and 250 μM peptide solutions. b, g) Cumulative release after 8 h from b) BC and g) TC dressings. c, h) Percentage release of PLCN8 αβ from c) BC and h) TC dressings after 8 h incubation. d, i) Mass of PLNC8 αβ released per mm^2^ of the dressing, for d) BC and i) TC dressings. e, j) Cumulative release simulating an exudating wound, where half the incubation volume was replaced every 2 h with fresh PBS +10 % FBS over 8 h for e) BC dressings and j) TC dressings. All data were obtained with n = 3.

The effective targeting and elimination of bacteria in the wound relies on the release of PLNC8 αβ from the wound dressings. Since the antimicrobial activity of PLNC8 αβ is closely linked to the combined interaction of both peptides with the bacterial membranes, achieving a release profile resulting in a 1:1 peptide ratio is advantageous. The spontaneous desorption and release of PLNC8 αβ from BC and TC dressings was studied for all loading conditions over an 8 h period in PBS +10 % FBS to mimic wound fluid. Interestingly, the peptide release kinetics differed substantially for the two materials. Peptides adsorbed in BC demonstrated a burst release during the first 1–2 h, followed by a more sustained release for PLNC8 α (Fig. 7a) resulting in a cumulative release of about 34 % of the loaded peptides over an 8-h time period corresponding to a concentration of 44 μM for the highest loading condition (250 μM) (Fig. 7b and c). The resulting PLNC8 α to PLNC8 β ratio (∼1.4–1.7) was slightly higher than desired. Whereas the burst release is likely an effect of shifting the equilibrium below the CMC, the slower release observed after the burst phase is probably a combined effect of the relatively low peptide solubility and interactions between the peptides and the BC fibrils. However, when normalized with respect to dressing area, as much as 1.4 ± 0.3 μg/mm^2^ of PLNC8 α and 0.7 ± 0.1 μg/mm^2^ of PLNC8 β was released after 8 h, following 250 μM PLNC8 αβ loading (Fig. 7d). To simulate peptide release from a highly exudative wound, the incubation solution was iteratively replaced with new PBS +10 % FBS, which was expected to result in more pronounced release due less pronounced rebinding of the peptides. Under these circumstances, the release of PLNC8 α released from BC dressings increased for all conditions, despite remaining below MIC, with the highest release (9 μM for PLNC8 α and 4 μM for PLNC8 β) being recorded on the second incubation for dressings loaded with 250 μM.

The release of the peptides from the TC dressings showed a similar burst behavior as for the BC dressings (Fig. 7f). However, no sustained release was seen for neither of the two peptides, which likely is an effect of the higher charge density of the TC. For all loading concentrations, PLNC8 β was released to a larger extent than PLNC8 α resulting in a PLNC8 α to PLNC8 β ratio of 0.52–0.44 when loaded using concentrations ranging from 25 to 250 μM after 8 h (Fig. 7g). Cumulative release after 8 h did not exceed 13 % for PLNC8 α and 20 % for PLNC8 β of the total amount of loaded peptides, confirming the strong electrostatic interaction between the TC and the peptides (Fig. 7h). Peptide release from TC dressings loaded with 250 μM PLNC8 α and β equaled 0.6 ± 0.1 μg/mm^2^ and 1.5 ± 0.2 μg/mm^2^, respectively, (Fig. 7i). When replacing the buffer with fresh buffer (PBS +10 % FBS) every 2 h to simulate a high exudate wound, the release of PLNC8 β increased but remained below MIC, (Fig. 7j).

### 2.6 Antimicrobial properties of BC/TC loaded with PLNC8 αβ

**Fig. 8:**
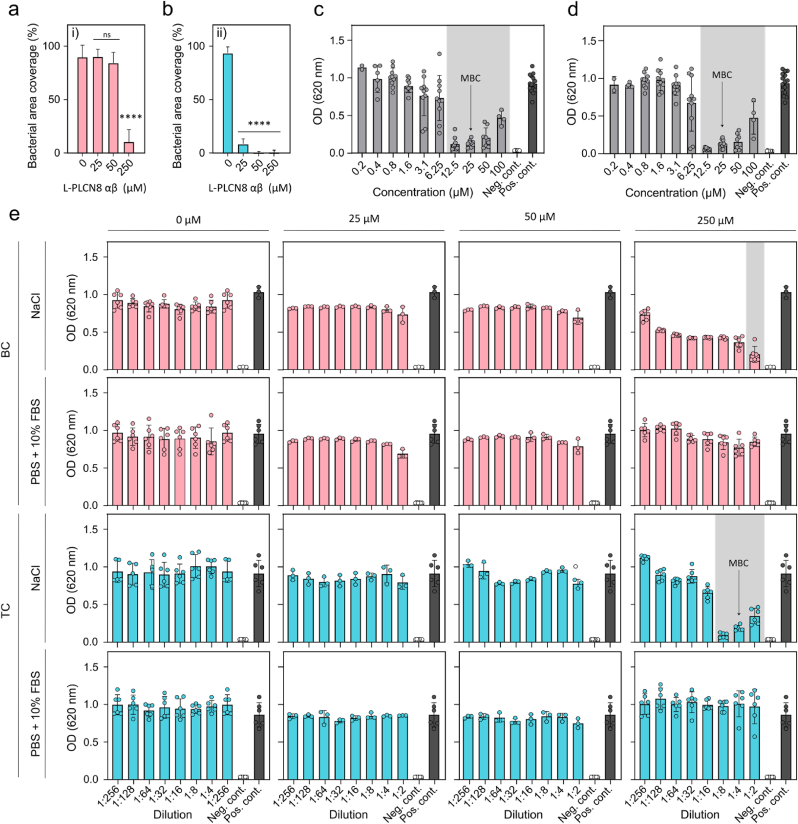
*S. aureus* coverage (%) on surface of loaded dressings after direct inoculation and 24 h incubation of a) BC- and b) TC-loaded dressings. ∗∗∗∗ = P < 0.0001 (n = 3). Broth microdilution test of PLNC8 αβ in c) Saline solution (0.9 % NaCl) and d) PBS + 10 % FBS. Shaded area indicates visible MIC. e) Broth microdilution of BC and TC dressings loaded with 0 μM (control), 25 μM, 50 μM and 250 μM PLNC8 αβ Shaded area indicates visible MIC.

To evaluate the antimicrobial properties of the PLNC8 αβ loaded BC/TC dressings, 6 mm ø pieces of the dressings were placed on an MH-agar plate, covered with *S. aureus* inoculate (∼10^5^ CFU/mL) and incubated at 37 °C overnight. *S. aureus* readily grew on both dressings without PLNC8 αβ, resulting in almost 100 % bacterial overgrowth after 24 h (Fig. 8a and b), confirming that the unfunctionalized dressings did not possess any antibacterial properties. The BC dressings loaded with 250 μM PLNC8 αβ showed almost complete inhibition of bacterial growth, whereas the two lower concentrations showed no significant inhibition (Fig. 8a). Interestingly, the TC dressings showed significant inhibition of bacterial growth for all PLNC8 αβ concentrations (Fig. 8b). A broth microdilution experiment was conducted to further investigate the effect of released peptides on bacteria cultured in suspension. As per the protocol for the release kinetics, dressings (ø 12 mm), with or without PLNC8 αβ, were incubated in 250 μl of either saline solution or PBS + 10 % FBS overnight. The dressings were then removed and *S. aureus* diluted in LB-broth was added to the wells and the optical density (OD) was measured over time. As a control, PLNC8 αβ was added directly to the bacteria cultures, which resulted in a MIC and MBC of 12.5 and 25 μM, respectively (Fig. 8c and d). Both BC and TC dressings loaded with 250 μM PLNC8 αβ showed efficient inhibition of bacterial growth, however, only PLNC8 αβ loaded TC dressings incubated in saline solution reached the MBC.

### 2.7 Fabrication of BC-MSN and TC-MSN

**Fig. 9:**
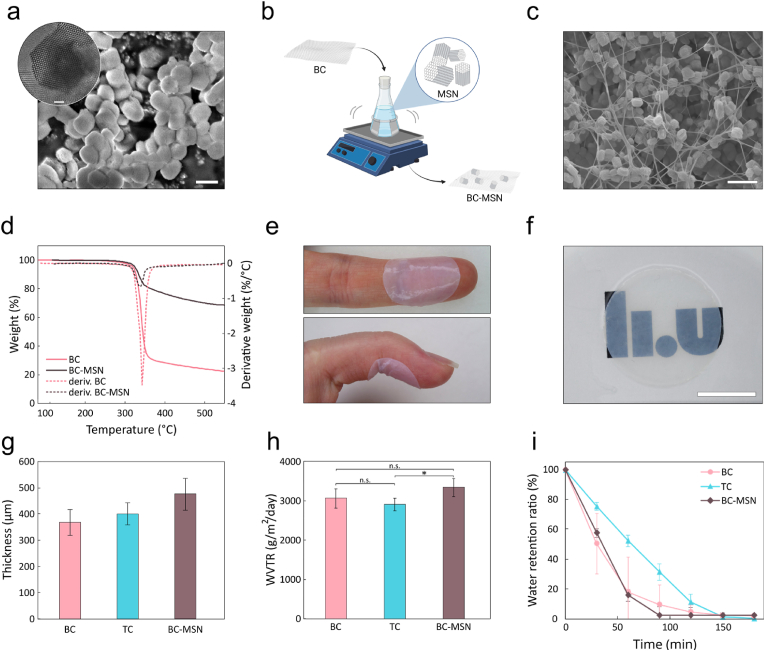
a) SEM micrographs (scale bar: 500 nm) and TEM micrographs (scale bar: 50 nm, in inset) of MSN SBA-15. b) Schematics of BC/TC-MSN composite production process. c) SEM micrograph of BC-MSN composite (scale bar: 500 nm). d) TGA curves of pristine BC and BC incubated in 5 mg/mL MSN for 5 days (BC-MSN), n = 10 e) conformability of BC-MSN dressings on intact skin, f) transparency of BC-MSN dressings (scale bar 1 cm). g) thickness of BC, TC and BC-MSN samples (n ≥ 6), h) Water vapor transmission rate (WVTR) of BC, TC and BC-MSN dressings. Results not significant at p < 0.01 (n ≥ 4). i) Water retention ratio (WRR) of BC, TC and BC-MSN dressings (n ≥ 5).

To increase the peptide loading capacity of the dressings, and thus improve on their antimicrobial properties, we explored the possibilities to functionalize the dressings with mesoporous silica nanoparticles (MSN of SBA-15 type) [66]. MSNs have been extensively used for drug delivery and have demonstrated promising biocompatibility and safety profiles, making them attractive candidates for biomedical applications, including drug delivery and medical device integration [67,68]. The size of the MSNs was about 400 nm with ordered hexagonal mesopores (Fig. 9a) with a pore size of about 11 nm (Fig. S9b). Owing to the large pore volume (1.00–1.15 cm^3^/g), a specific surface area of 600–700 m^2^/g was achieved (Fig. S9a), which in combination with the negatively charged silanol groups present on both their internal and external surface, could facilitate loading of large amount of PLNC8 αβ. Synthesis of MSNs in the presence of BC resulted in the production of a fragile composite material presenting a wide range of MSN shapes (Fig. S10a). The low mechanical stability of the material was most likely an effect of the thermal treatment required to remove the surfactant from the MSN mesopores, which resulted in degradation of the nanocellulose network (Fig. S10b) and a low number of MSNs in the dressings (Fig. S10c). To avoid this issue, we instead investigated the possibility to assemble colloidal MSNs in the dressings by tuning the interaction potential between the MSNs and the nanocellulose fibrils by modulating the ionic strengths of the suspensions [51,62]. The BC and TC dressings were incubated in a suspension of SBA-15 (1–5 mg/mL) for up to 5 days under agitation (Fig. 9b). Despite using identical conditions, the result of the MSN assembly process was very different for BC and TC. In BC, the MSNs were able to penetrate the nanocellulose network and adhere to the fibrils throughout the dressings as indicated by SEM (Fig. 9c) resulting in the loading of 6.8 μg MSN/mm^2^ BC (Fig. 9d and Fig. S11a) and in an increase of specific surface area from 88 m^2^/g of BC to 265 m^2^/g [51]. The BC-MSN composite material retained excellent conformability with no obvious differences to pristine BC (Fig. 9e). However, the light scattering by the colloidally sized MSNs made the MSN functionalized BC dressings slightly less transparent compared to pristine BC (Fig. 9f). By increasing the incubation time (Fig. S11b) and/or MSN concentration (Fig. S11c), the amount of adsorbed MSNs could be increased, resulting in higher loading capacity but a swelling and a decrease in mechanical flexibility of the dressing compared to pristine BC. BC functionalized with 5 mg/mL MSN for 5 days displayed a similar thickness as pristine BC and TC dressings (Fig. 9g) and high flexibility, which contributed to high conformability and was thus considered as a good concentration for further studies.

Functionalization of BC with MSNs had no statistically significant effect on the water vapor transmission rate (WVTR) of the dressings, with an estimated WVTR of 3336 ± 235 g/m^2^/day compared to 3058 ± 241 g/m^2^/day for BC and 2907 ± 160 g/m^2^/day for TC [49] (Fig. 9h), which is close to the reported optimal WVTR range of 2000–2500 g/m^2^/day [69] for wound healing. Moreover, high moisture retention of the dressings can reduce scarring, frequency of dressing changes, and healing time, while ensuring a pain-free healing [70]. Moisture retention was assessed by measuring water retention ratio (WRR). Both BC and BC-MSN showed slightly lower water retention (90–120 min) than TC (150 min), which most likely is due to the higher degree of swelling of the TC dressings. Notably, WRR of pristine BC dressings displayed high deviations, due to the inhomogeneity in dressing thickness (Fig. 9i).

Functionalization of TC with MSNs turned out to be more challenging. Whereas the BC dressings presented a consistent MSN loading even at the lowest MSN concentration (1 mg/mL, Figs. S12a and b), this was not seen for the TC dressings (Fig. S12c). SEM images confirmed a low number of adsorbed MSNs in the TC dressings, even for the highest MSN concentration (5 mg/mL) tested (Fig. S12d). In TC, the MSNs appeared to adsorb primarily at the surface of the dressings. We hypothesized that the density of the fibrillar nanocellulose network structure and the lower porosity of TC dressings could hinder MSN penetration into the material. In addition, the high charge density and small nanofiber widths (1.9 ± 0.9 nm) of TC [49], compared to BC [62], most likely contribute to charge repulsion exceeding the attractive van der Waals interaction that promote the self-assembly process seen in BC [49]. To investigate if we could circumvent this issue, we also investigated the possibility to produce TC-MSN dressings by homogenizing MSN nanoparticles in the TC gel prior to hydrogel formation by solvent casting. Hydrogels were prepared with equal nanocellulose grammage (20 g/m^2^) to pristine TC dressings. However, the stability of the dressings was seen to be significantly influenced by the presence of MSNs. Whereas dressings produced with 0.3 wt% and 2.9 wt% MSN had similar mechanical properties as pristine TC, higher MSN concentrations (on par with BC-MSN) influenced the nanocellulose network formation and resulted in poorly cohesive and brittle materials (Fig. S13a). SEM micrographs of the dressings confirmed that the MSNs were well dispersed when using low concentrations of MSNs (Figs. S13b and c), whereas higher MSN concentrations resulted in aggregation of the MSNs in the TC matrix (Fig. S13d). Surface functionalization or alternative incorporation methods could potentially improve MSN loading efficiency in TC dressings. However, due to the challenges to obtain TC-MSN composites with similar concentrations of MSNs as for the BC dressings, we focused the remaining of the work on BC-MSN.

### 2.8 PLNC8 αβ loading in suspended MSNs

**Fig. 10:**
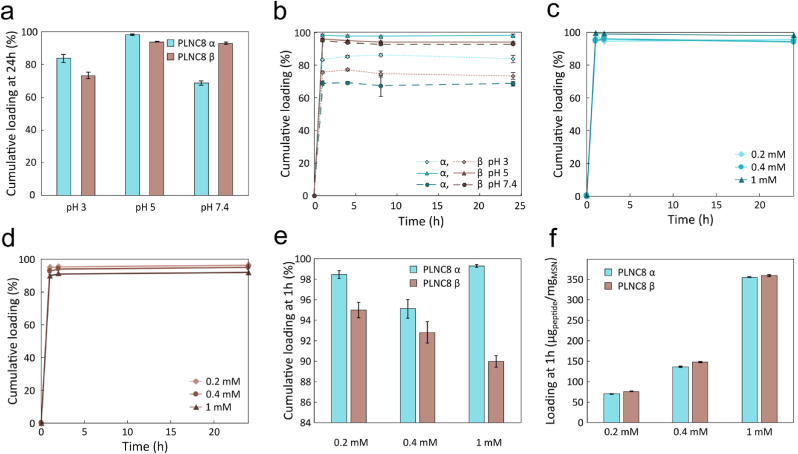
a-b) Cumulative loading of peptide 200 μM PLNC8 αβ in MSN, at pH 3, pH 5, pH 7.4: a) cumulative loading at 24 h, and b) loading kinetics over 24 h. c-d) Loading of c) PLNC8 α and d) PLNC8 β in MSN (n = 3). e) Cumulative loading at 1 h of PLNC8 αβ at pH 5 expressed as e) loading efficiency (%) and f) mass_peptide_ per unit mass_MSN_ (n = 3).

Prior to loading of PLNC8 αβ in BC-MSN, the loading was first evaluated in suspended SBA-15 MSNs. Loading was tested for both peptides at pH 3, pH 5, pH 7 to optimize the interactions between the cationic peptides and the negatively charged MSNs. The positive net-charge of PLNC8 α decreases from +6 at pH 3 to +4 at pH 7.4 whereas the net-charge of PLNC8 β decreases from +8 at pH 3 to +5 at pH 7.4. Likewise, increasing the pH from 3 to 7 results in a more negative zeta potential of the MSNs. The peptides (200 μM) were incubated with MSNs (10 mg/mL) for 24 h. The highest loading was observed at pH 5, resulting in about 98 % of PLNC8 α and 94 % of PLNC8 β retained in the MSNs (Fig. 10a). The loading was rapid (<1 h) and no significant differences in loading kinetics were seen when varying the pH from 3 to 7 (Fig. 10b). Increasing the peptide concentration from 200 μM to 400 and 1000 μM during loading did not have any dramatic effect on the loading kinetics and the loading efficiency was >95 % for PLNC8 α and >90 % for PLNC8 β after 1 h for all concentrations at pH 5 (Fig. 10c–e). The mass of loaded peptides was proportional to the peptide concentration in solution, with PLNC8 α/β encapsulation being as high as 356 ± 0.5 μg/mg_MSN_ for PLNC8 α and 360 ± 2 μg/mg_MSN_ for PLNC8 β for the highest loading concentration (Fig. 10f).

### 2.9 PLNC8 αβ release from suspended MSNs

**Fig. 11:**
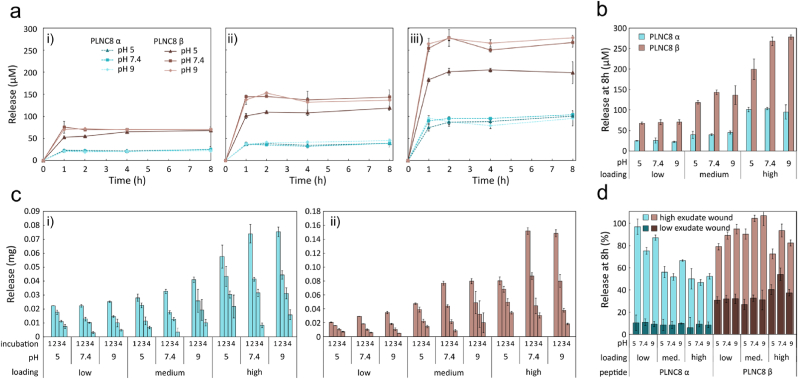
a) Release of peptide PLNC8 αβ from MSNs in PBS+10 % FBS (4 mg/mL) at pH 5, 7.4 and 9, when peptide was loaded at i) low concentration (0.2 mM), ii) medium concentration (0.4 mM) and iii) high concentration (1 mM), n = 3. b) Cumulative release at 8 h (same conditions as (a)). c) Peptide i) PLNC8 α and ii) PLNC8 β release from MSN (4 mg/mL in PBS+10 % FBS) simulating a high exudate wound condition, where every 2 h half of the volume of the release solution was replaced with fresh buffer (corresponding to incubation 1–4), n = 3. d) Percentage released PLNC8 αβ from MSNs under low and high exudate wound conditions after 8 h incubation (n = 3).

PLNC8 αβ release from peptide loaded MSNs (4 mg/mL) was evaluated separately for the two peptides over a period of 8 h in PBS+10 % FBS at pH 5, pH 7.4 and pH 9 at 37 °C, representing the pH values typically seen in non-infected and infected wounds [[71], [72], [73]]. A burst release was seen for all pH values for all loading concentrations with a plateau reached within 1 h. The release was most pronounced at high pH values. Despite similar amount of peptides loaded, the release of PLNC8 α was less extensive than for PLNC8 β (Fig. 11a and b), resulting in a final PLNC8 α and PLNC8 β concentration of 95–103 μM and 199–278 μM, respectively, at the highest loading condition, and a PLNC8 α to PLNC8 β ratio of 0.34–0.51. No further peptide release was seen during the 8 h period, indicating that equilibrium was reached. By simulating the release conditions in a high exudate wound by replacing half of the buffer every 2 h, the equilibrium was shifted, causing a more sustained peptide release (Fig. 11c and d).

### 2.10 PLNC8 αβ loading and release from BC-MSN

**Fig. 12:**
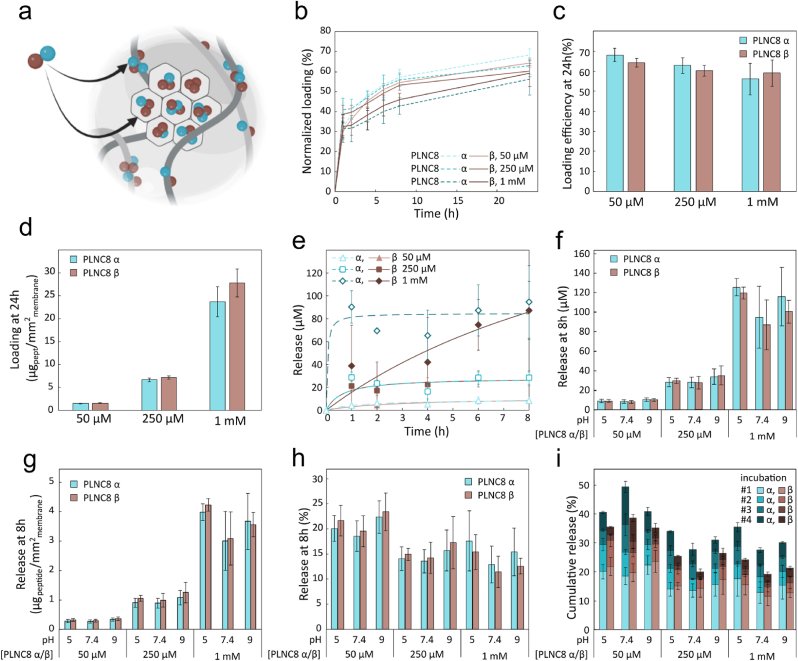
a) Schematic of PLNC8 αβ loading in BC-MSN composites. b) Cumulative loading kinetics, c) loading efficiency at 24 h and d) loaded peptide mass per unit dressing area (at 24 h) of peptides PLNC8 αβ in BC-MSN composites (prepared by incubating BC dressings in 5 mg/mL MSN for 5 days). Three concentrations were investigated: 50 μM, 250 μM and 1 mM PLNC8 α/β (1:1) in milliQ water, pH 5 (n = 9). e) PLNC8 αβ release kinetics from BC-MSN dressings, representative curve at pH 7.4 (n = 3). f) PLNC8 αβ release concentration at 8 h, g) mass per unit area and h) percentage at 8 h incubation at pH 5, 7.4 and 9 (n = 3). i) Cumulative PLNC8 αβ release simulating exudative conditions, where half the incubation volume was replaced every 8 h with fresh one, for a total of four incubations (n = 3).

After confirming the possibility to load and release relevant concentrations of PLNC8 αβ from suspended MSNs, we investigated the possibility to use BC-MSN to increase the concentrations of released PLNC8 αβ to improve the antimicrobial properties of the dressings (Fig. 12a). BC-MSN dressings with about 6.7 μg of MSN per mm^2^ were loaded with 50 μM, 250 μM or 1 mM PLNC8 αβ (1:1) and the loading kinetics was evaluated for a period of 24 h. After 1 h, 32–41 % of PLNC8 α and 30–39 % of PLNC8 β had been adsorbed in the dressings. After 24 h, 56–68 % and 59–64 % of the PLNC8 α and PLNC8 β had been adsorbed in the dressings, respectively (Fig. 12b and c). Compared to pristine BC (Fig. 6c), the presence of MSNs resulted in a more equal loading of PLNC8 α and β. By increasing the peptide concentration to 1 mM, we could obtain a loading as high as 23.7 ± 3.3 μg/mm^2^ of PLNC8 α and 27.9 ± 3.1 μg/mm^2^ of PLNC8 β while still maintaining a PLNC8 α to PLNC8 β loading ratio of 0.83 (Fig. 12d).

A burst release profile was seen for both peptides loaded in BC-MSN (Fig. 12e). The cumulative release at 8 h was primarily dependent on the loading concentration and a peptide concentration of about 125 μM was achieved when the dressings were loaded with 1 mM (Fig. 12f) with a PLNC8 α to β ratio close to 1:1 (Fig. 12g). Whereas pristine BC displayed a PLNC8 α to PLNC8 β release ratio of 1.4 (50 μM)-1.7 (250 μM) (Fig. 7b), BC-MSN showed a release ratio of 0.99–1 for the same experimental conditions. The total release did not surpass 24 % (50 μM loading), 17 % (250 μM loading) and 18 % (1 mM loading) for each peptide during the initial 8 h, highlighting the possibility to rapidly reach MBC values while enabling long-term release as a result of MSN degradation. By simulating high exudate wound conditions, the shift in the equilibrium resulted in additional peptide release (Fig. 12i, Fig. S14).

### 2.11 Antimicrobial properties of BC-MSN loaded with PLNC8 αβ

**Fig. 13:**
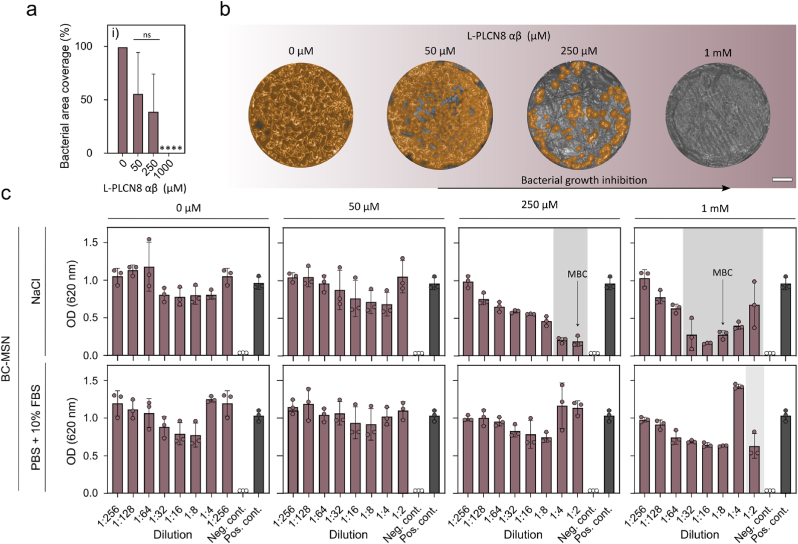
a) *S. aureus* coverage (%) on surface ofPLNC8 αβ loaded dressings after direct inoculation and 24 h incubation of BC-MSN-loaded dressings. ∗∗∗∗ = P > 0.0001 (n = 3) and b) false-colored stereomicroscopy photos of the same (scale bar: 2 mm). The colored structures correspond to: orange: *S. aureus,* grey: dressing. c) Broth microdilution of BC-MSN dressings loaded with 0 μM (control), 50 μM, 250 μM and 1000 μM PLNC8 αβ. Shaded area indicates visible MIC. (For interpretation of the references to color in this figure legend, the reader is referred to the Web version of this article.)

BC-MSN loaded with 0, 50, 250 or 1000 μM PLNC8 αβ placed on MH-agar plates, covered with *S. aureus* inoculate (∼10^5^ CFU/mL) and incubated at 37 °C overnight showed complete inhibition of bacterial growth for the highest concentration, and approximately 50 % inhibition for the two lower concentrations. Dressings functionalized with MSN but without the addition of PLNC8 αβ showed no bacterial inhibition (Fig. 13a). A broth microdilution assay showed improved bacterial inhibition for PLNC8 αβ loaded BC-MSN compared to BC and TC loaded with PLNC8 αβ by physisorption (Fig. 13b). For the higher loading concentrations, MBC was reached in saline but not in PBS + FBS, which likely is an effect of the lower solubility of the peptides in the LB culture broth in combination with protein corona formation that restricted the diffusion of peptides from the MSN pores. However, the low solubility of the peptide did not impede their antimicrobial activity in the infected porcine wounds indicating that other factors in the wound microenvironment could impact bioavailability, including pH, protein degradation products, and ECM components [9,74]. Moreover, on slightly longer time scales (>8 h), MSN degradation will result in release of all of the loaded peptides [75], resulting in concentrations that vastly exceed MIC, indicating that the BC-MSN strategy is a promising carrier and delivery system for PLNC8 αβ.

### 2.12 Cytocompatibility of PLNC8 αβ loaded dressings

**Fig. 14:**
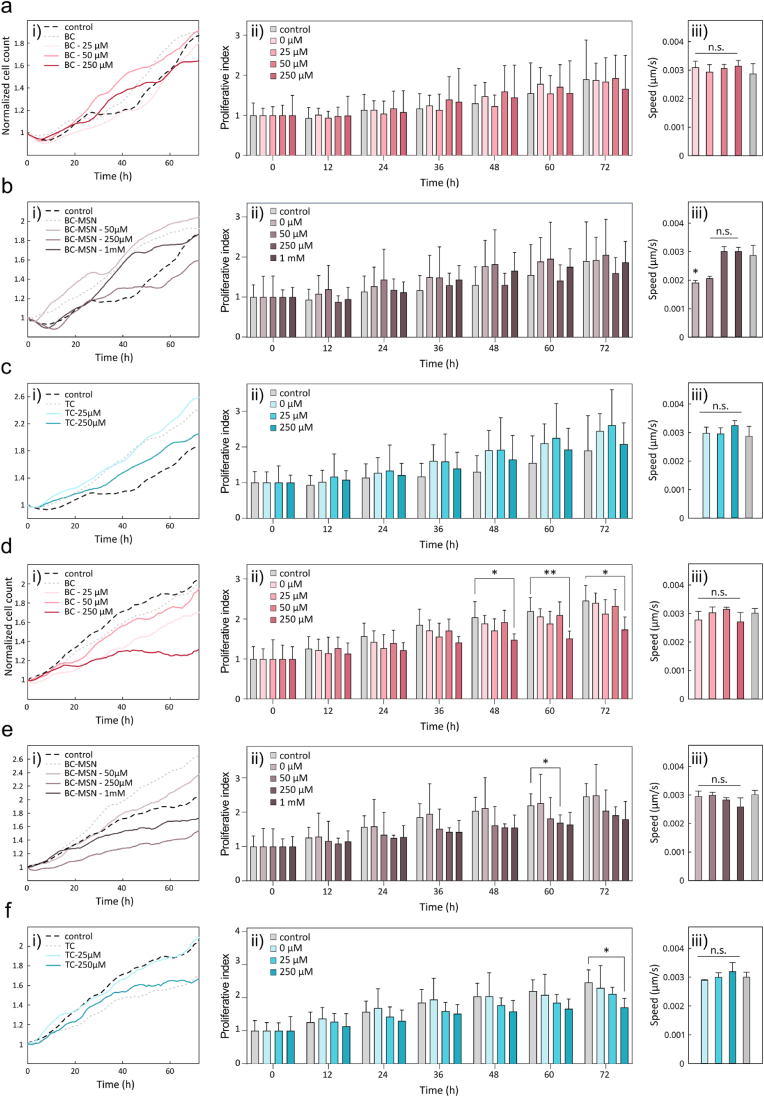
Cytocompatibility testing of a,d) BC, b,e) BC-MSN and c,f) TC wound dressings without (control) and with PLNC8 αβ (25 μM - 1 mM) on a-c) human primary keratinocytes (n ≥ 7) and d-f) human primary fibroblasts (n = 4). i) Normalized cell count analysis over 72 h when cultured in 10 % eluates from wound dressings, results displayed as mean. ii) Normalized cell count analysis at relevant timepoints over 72 h culture. Results displayed as mean and standard deviation. Statistical analysis was carried on using a two-way ANOVA complemented with Dunnett's multiple comparison test (∗P < 0.1, ∗∗P < 0.01, n.s. whereas not indicated). iii) Cell migration data displayed as mean and standard deviation. Statistical analysis was carried on using a one-way ANOVA complemented with Dunnet's multiple comparison test (∗P < 0.1).

The cytocompatibility of PLNC8 αβ loaded wound dressings was assessed using human primary keratinocytes and fibroblasts. Peptide-loaded BC, TC, and BC-MSN dressings were incubated in cell culture medium for 8 h, after which the eluates were applied to the cells. Cell proliferation and migration were continuously monitored over 72 h. Treatment with the dressings did not significantly affect the proliferation of human primary keratinocytes compared to untreated controls for any of the dressing types at 72 h (Fig. 14a–c). Growth curve analysis revealed that cells exposed to both loaded and unloaded TC dressings exhibited an accelerated growth rate relative to the control (Fig. 14c). For human primary fibroblasts, a similar trend in proliferation was observed, except for BC dressings loaded with 250 μM PLNC8 αβ, which exhibited a reduction in fibroblast proliferation. All BC-based dressings, particularly those loaded with the highest peptide concentration (250 μM), induced a decrease in fibroblast proliferation compared to the untreated controls (Fig. 14d). Cell migration dynamics were also evaluated throughout the experimental period. Keratinocyte motility decreased by 36 % and 28 % upon exposure to unloaded BC-MSN and BC-MSN loaded with 50 μM peptide, respectively (Fig. 14b). Fibroblast migration showed a decrease of 12 % and 15 % compared to the control after exposure to BC and BC-MSN oaded with 250 μM peptide (Fig. 14d–iii,e-iii). The reduction in fibroblast proliferation and migration, particularly at higher peptide concentrations, could warrant further investigation. However, the overall response indicate that the peptide-loaded dressings had no major adverse effects on human primary keratinocytes and fibroblasts, indicating that the dressings could provide an attractive strategy for *in vivo* delivery of high concentrations of PLNC8 αβ for treatment of wound infections.

## 3 Conclusions

In this study, we evaluated the efficacy of the protease resistant two-peptide bacteriocin PLNC8 αβ for the treatment of wound infections and developed strategies to optimize the controlled of release PLNC8 αβ from advanced nanocellulose-based wound dressings. Both the L- and D-enantiomers of PLNC8 αβ were assessed using human *in vitro* models and an infected porcine *in vivo* wound model. D-PLNC8 αβ showed the highest efficacy, resulting in complete eradication of the infection. Administration of L-PLNC8 αβ resulted in a significant reduction in bacterial load but the wounds remained infected. However, co-administration of L-PLNC8 αβ with gentamicin decreased the bioburden to levels comparable to normal flora, due to synergistic effects. The difference in efficacy between the enantiomers is likely due to the proteolytic stability of the D-peptide. Despite the high protease activity in infected wounds, D-PLNC8 αβ can consequently remain intact and functional. Moreover, the potent antimicrobial activity of D-PLNC8 αβ and the synergistic interaction between L-PLNC8 αβ and gentamicin present interesting therapeutic possibilities for treatment of wound infection that can reduce the need for conventional antibiotics. Both peptides were well-tolerated by human primary fibroblasts and keratinocytes *in vitro* and promoted wound re-epithelialization *in vivo*. To facilitate their clinical application, we explored strategies for delivery of PLNC8 αβ using nanocellulose-based wound dressings as carriers for topical localized and sustained peptide release. Two wound dressing platforms were examined: a commercially available biosynthetic nanocellulose dressing (BC, Epiprotect™) and a BC-mimicking dressing derived from TEMPO-oxidized hardwood nanocellulose (TC). The dressings could conform to the irregularities of the skin and wounds and support movement and created a moist, protected wound environment conducive to healing. The PLNC8 αβ functionalization of BC and TC dressings was initially explored by physisorption of the peptides onto the nanocellulose fibrils. Both the PLNC8 αβ functionalized BC and TC dressings were effective at inhibiting bacterial growth on their surfaces but were less efficient in inhibiting bacteria in suspension. To enhance the peptide-loading capacity to enable loading and release of higher concentrations of peptides, BC dressings were functionalized with mesoporous silica nanoparticles (MSNs). The MSN functionalization significantly increased the surface area of the dressings from 88 to 265 m^2^/g. MSN functionalization of TC was more complicated and will require additional optimization. Importantly, the BC-MSN composite maintained key wound dressing properties, including conformability, transparency, and moisture management, while also improving peptide loading capacity and achieving a more balanced release of the PLNC8 α and β peptides. BC-MSN demonstrated high loading efficiencies, with over 90 % of the peptides adsorbed within 1 h. Peptide release was rapid and dependent on the simulated wound volume. BC-MSN dressings exhibited enhanced antimicrobial activity against *S. aureus*, offering the potential for effective infection control.

Although BC dressings are already produced at industrial scale and are adopted in the clinic, the production is relatively time consuming and expensive. TC dressings represent an interesting and cost-effective alternative to BC. The scaling up of TC production remains a technical challenge but could likely leverage on existing strategies from the paper making industry, which would significantly lower the production costs of advanced nanocellulose wound dressings. Future studies will focus on evaluating long-term wound healing outcomes, effects on scar formation, and the local tissue effects of MSN degradation to further assess the safety and translational potential of the antimicrobial dressings. In summary, D-PLNC8 αβ demonstrate high antimicrobial activity *in vivo* and the combination of PLNC8 αβ with advanced nanocellulose-based dressings, particularly those functionalized with MSNs, represents a promising approach for the localized treatment of wound infections. The functionalized dressings demonstrated a significant potential to reduce bacterial bioburden in infected wounds, offering a new generation of antimicrobial wound therapies and possibilities to combat persistent multidrug-resistant wound infections.

## 4 Methods

***General:*** All chemicals were bought from Merck Life Science AB (Stockholm, Sweden) unless otherwise stated. Epiprotect™ basic (EPBASIC50) bacterial cellulose dressings used in this study were supplied by S2Medical AB (Linköping, Sweden). Hardwood (Aspen, SweTree Technologies, Umeå, Sweden) powder was used in production of TC dressings as previously described [49]. TC dressings were supplied in dry form and rehydrated before use.

**Peptide synthesis:** All amino acids were obtained from Merck Life Science AB (Stockholm, Sweden). The peptides PLNC8 α (H_2_N-DLTTKLWSSWGYYLGKKARWNLKHPYVQF-COOH) and PLNC8 β (H_2_N-SVPTSVYTLGIKILWSAYKHRKTIEKSFNKGFYH-COOH) with free C-terminals (-COOH) and N-terminals (-NH_2_) were purchased from GL Biochem (Shanghai) Ltd. or synthesized in house. The synthesis was performed using conventional Fmoc chemistry on a Liberty Blue automated microwave peptide synthesiser (CEM corporation, Matthews, United States) in a 0.1 mmol scale. Cl-MPA ProTide (LL) high swelling resin (CEM corporation, 0.16 mmol/g) was used as solid support, and chloride loading of the first amino acids (10 equivalents (eq)) was performed with addition of N,N-Diisopropylethylamine (DIEA, 20 eq) and potassium iodide (KI, 2.5 eq). Peptide coupling was performed using a 5-fold excess of amino acids (Iris Biotech GmbH, Marktredwitz, Germany), a coupling temperature of 90 °C and coupling time of 4 min. Special coupling cycles were employed for Histidine residues (double coupling at 50 °C, for 10 min), to minimize epimerization. Single coupling was performed for conjugation of the first 10 residues while double coupling was performed for the subsequent residues, to increase the yield of the reaction. Activator DIC (5 eq) and activator base Oxyma (5 eq) were employed. Fmoc deprotection of coupled amino acids was performed via treatment with Piperidine (20 % in DMF, v/v). The peptides were cleaved from the solid support and globally deprotected via treatment with a mixture of trifluoroacetic acid (TFA)/triisoproylsilane (TIS)/water (95:2.5:2.5, v/v/v) for 2 h. The peptides were filtered, concentrated with a flow of N_2_, and precipitated twice in cold diethyl ether. Crude peptides were purified on a C18 reversed phase column (ReproSil Gold 120C18, Dr. Maisch GmbH) attached to a semipreparative HPLC system (Dionex UltiMate 3000, Thermo Scientific) using an aqueous gradient of acetonitrile (10 %–50 %) containing 0.1 % TFA. Purity was confirmed using a XBridge BEH Phenyl Hexyl column (Waters Corporation, Milford, United States, Fig. S3). Mass identity of the peptides was confirmed by MALDI-TOF Mass Spectrometer (UltrafleXtreme, Bruker Daltonics, Billerica, United States) using α-cyano-4-hydroxycinnamic acid as matrix (Fig. S4). Peptide net charge was calculated through the software PepCalc.com (Innovagen AB, Lund, Sweden).

**Isolation of human primary keratinocytes and fibroblasts:** Cells derived from healthy patients undergoing routine reduction surgeries at Linköping University Hospital, Sweden, performed under ethical approval from the Swedish Ethical Review Authority (2018/97/31). Isolation was conducted according to a modified protocol described by Rheinwald and Green. [76] Briefly, the remaining tissue, upon mechanical removal of subcutaneous fat, was enzymatically digested overnight. [76] Keratinocytes were isolated from the epidermis in Dulbecco's modified eagle medium (DMEM; Gibco Thermo Fisher Scientific, Paisley, UK) containing 2.5 mg mL^−1^ dispase (Gibco Thermo Fisher Scientific) with a 4 °C overnight incubation. Fibroblasts were isolated from the dermis in DMEM containing 165 U/mL collagenase (Gibco Thermo Fisher Scientific) and 2.5 mg mL^−1^ dispase with an incubation at 37 °C, 5 % CO_2_, and 95 % humidity. Both cell types were thereafter incubated for 15 min in DMEM containing 0.02 % versene/0.1 % trypsin (Gibco Thermo Fisher Scientific). Once isolated, keratinocytes were seeded into 75 cm^2^ culture flasks (Falcon Corning Inc, Corning, USA) with Keratinocyte Serum-Free Medium (KSFM; Gibco Thermo Fisher Scientific) containing 1 mg L^−1^ epidermal growth factor, 25 mg L^−1^ bovine pituitary extract, 50 U mL^−1^ Penicillin and 50 mg mL^−1^ Streptomycin (Gibco Thermo Fisher Scientific). Fibroblast were seeded into 75 cm^2^ culture flasks with DMEM containing 10 % Fetal Calfe Serum (FCS; Gibco Thermo Fisher Scientific), 50 U mL^−1^ Penicillin and 50 mg mL^−1^ Streptomycin. Medium was changed thrice weekly until confluency was reached.

**Toxicological risk assessment of PLNC8 αβ:** Cytotoxicity of L- and D-PLNC8 αβ was performed on human primary keratinocytes and fibroblasts. Following expansion of primary cultures, cells were enzymatically detached using 0.02 % versene/0.1 % trypsin) and seeded in flat-bottom 96-well plates (Falcon, Corning Inc). Keratinocytes were allowed to adhere for 48 h and fibroblast for 24 h before treatment with L- and D-PLNC8 αβ in various concentrations (n=2-3). Data was collected using a LiveCyte 2 kinetic cytometer (Phase Focus Ltd, Sheffield, UK), in which cells were continuously monitored for 72 h at 37 °C, 5 % CO_2_, and 95 % humidity. Ptychographic images were captured every hour. Data was exported and analyzed using PF Assay Analysis v.3.7.1 (Phase Focus Ltd). Cell count for all treatment groups was exported to Prism 8.0 (Graphpad, LaJolla, US) for statistical analysis and generation of graphs. Cell counts were normalized to non-treated controls and expressed as a proliferative index. Average cell speed for each well was expressed as μm s^−1^. Cell proliferation and speed were compared using a two-way ANOVA coupled with a Dunnett post-test. All values are plotted as mean ± standard deviation. P values < 0.05 were considered statistically significant.

**Bacterial culture:***S. aureus* (ATCC 29213, MSSA, ATCC, Manassas, VA), were streaked on Müller-Hinton (MH) agar plates and incubated at 37 °C overnight. Prior to the experiments, single colonies were inoculated into 5 mL of LB broth and incubated at 37 °C overnight. All bacteria were washed twice in PBS before use, and the CFU/mL determined by McFarland standards.

**Porcine in vivo infected wound model:** The antimicrobial efficacy of PLNC8 αβ was tested using a porcine *in vivo* wound model on four female pigs weighting between 40 and 50 kg. The study was conducted under approval from the Regional Animal Ethics Committee (ID 1418), adhered to the guidelines postulated by Linköping University, involved educated personnel and was supervised by a veterinarian. The animals were housed in boxes measuring 2 × 2.5 m at a state-of-the-art facility, with a light/dark circle of 12/12 h, an ambient temperature of 18–20 °C, daily feeding and free access to water and hay. All procedures were performed under general anesthesia, induced by intramuscular (IM) injection of 10 μg/kg dexmedetomidin (Dexdomitor; Orion Pharma, Danderyd, Sweden) and 3 μg/kg of tiletamine and zolazepam (Zoletil; Virbac, Kolding, Denmark). Animals were intubated with an endotracheal tube connected to an automatic ventilator. General anesthesia and analgesia were maintained with intravenous infusion of 3–7.5 mg/kg pentobarbital sodium (Pentobarbitalnatrium vet.; APL, Kungens Kurva, Sweden) in combination with 0.5–0.75 μg/kg fentanyl (Leptanal, Janssen, Solna, Sweden). Vital parameters were monitored by pulse oximetry, capnography and rectal thermometer. Signs of postoperative pain were treated with IM administration of 50–75 μg fentanyl and 40 mg meloxicam (Loxicom; N-Vet AB, Uppsala, Sweden). 20 circular full-thickness wounds with a diameter of 1 cm were created paravertebrally on each animal. All wounds were inoculated with 10^7^ colony forming units (CFU) Staphylococcus aureus (ATCC 29213, Manassas, VA, USA) and covered with occlusive (Tegaderm, Mölnycke, Sweden) and elastic dressings (Elastic bandage, Hansbo sport, Västra Frölunda, Sweden) two days prior to treatment. The experimental groups were randomly assigned to the wounds two days post inoculation. Wounds were treated with 1 ml of either saline solution, Gentamicin (10 μg/ml), L/D-PLNC8 αβ (300 μM), or a combination of Gentamicin (10 μg/ml) and L/D-PLNC8 αβ (100 μM). Wound biopsies were obtained on day 0, 2, 5 and 8. One half of the wound was resected with a scalpel and used for histological evaluation, and the other half was resected with a 3 mm punch biopsy (Paramount Surgical Ltd. Delhi, India) and used for quantitative bacterial cultures. After a maximum of ten days, the animals were euthanized by intravenous injection of 400 mg/kg pentobarbital sodium (Pentobarbitalnatrium vet.; APL, Kungens Kurva, Sweden) under anesthesia.

**Histological analysis of porcine wounds:** One biopsy per wound and timepoint was fixed in 4 % neutral buffered paraformaldehyde (Histolab Products AB, Gothenburg, Sweden) for 12 h, dehydrated through an ethanol–xylene series and embedded in paraffin. Samples were sectioned into 6 μm sections using a microtome (RM2255, Leica Biosystems, Wetzlar, Germany), mounted on slides (Epredia Superfrost Plus Adhesion Microscope Slides; Gerhard Menzel GmbH, Braunschweig, Germany) and stained using Hematoxylin and Eosin (Histolab Products AB) according to manufacturer's instructions. Coverslips (Gerhard Menzel GmbH, Thermo Fischer Scientific) were mounted on the stained slides using Pertex mounting medium (Histolab Products AB). Visualization was performed using a BX41 microscope (Olympus, Stockholm, Sweden) and images captured with a DP70 CCD camera (Olympus).

**Immunofluorescence analysis of porcine wounds:** A selection of the 6 μm sectioned samples were used for immunofluorescent staining of *S. aureus* colonies. Staining was performed according to manufacturer's instructions. Briefly, sections were rehydrated through a xylene-ethanol series prior to creation of hydrophobic barriers with a Liquid Blocker Super Pap-pen (Histolab Products Ab). Thereafter followed a blocking serum incubation at room temperature in humidity chambers. Positive controls were incubated with a primary polyclonal IgG *S. aureus* antibody (dilution 1:800, CAT #PA1-7246; Invitrogen, Thermo Fisher Scientific) at 4 *°C* overnight in a dark humidity chamber. Negative controls were incubated with PBS. Thereafter, the sections were incubated with a secondary anti-rabbit antibody (1:250, CAT #A11034, Invitrogen, Thermo Fisher Scientific) and 5 % DAPI nucleic stain (CAT #D1306; Invitrogen, Thermo Fisher Scientific). Coverslips (Gerhard Menzel GmbH, Thermo Fischer Scientific) were mounted on the stained slides using Prolong Glass Antifade Mountant (Thermo Fisher Scientific). Visualization was performed using a Leica Stellaris 5 confocal microscope (Leica Microsystems AB, Wetzlar, Germany) with the LAS X microscope software (Leica Microsystems AB).

**Quantitative cultures of porcine wounds:** One biopsy per wound and timepoint was used for quantitative cultures. Biopsies were stored at −20 °C and thawed on the day of culture. Thawed samples were weighted and mechanically homogenized by mincing in PBS. A dilution series of each sample was cultured in triplicates on Chapman agar. Plates were incubated overnight at 37 °C and bacterial colonies manually enumerated. CFU/g was generated through the mean value from the triplicate of each culture, multiplied with the dilution factor of the triplicate and divided with the biopsy weight.

**Nanocellulose dressing preparation:** Two different types of nanocellulose dressings were used for this study. Bacteria cellulose dressings (Epiprotect™, S2Medical AB, Linköping, Sweden), produced from *Komagataeibacter xylinus*, approximately 200 μm in thickness (7.6 g/m^2^), were stored in 70 % ethanol. The dressings were cut in 6 or 12 mm disks using a biopsy punch and washed in milliQ water for at least 30 min prior use. TEMPO-oxidized wood nanocellulose dressings were prepared as described in a previous study by Berglund et al. [49] Aspen wood underwent TEMPO-oxidation following a method previously described [77]. Briefly, NaClO_2_ (5 g/g dry wood) and TEMPO (17.5 mg/g dry wood) was added to a phosphate buffer (0.1 M, 100 ml/g dry wood, pH = 6.8). The flask was sealed and kept at a temperature of 60 °C for 72 h. Following the oxidation, the insoluble fractions were washed thoroughly with dH_2_O and a suspension with 0.2 wt% consistency was made, which then was nanofibrillated with a high-pressure homogenizer APV 2000 (SPX Flow Inc, Silkeborg, Denmark) at a 1000 Bar for 1 pass to obtain TEMPO-oxidized nanocellulose. The oxidation degree of the TEMPO-oxidized nanocellulose was determined by conductometric titration method, as previously described [49]; 50 mL of TEMPO-oxidized nanocellulose suspension (≈0.2 wt%) was diluted in 0.05 M HCl for protonation where starting pH of suspension was around 2–3. In, brief, conductometric titration was carried out with an Eco titrator (Metohm Nordic AB, Bromma, Sweden) using 0.01 M NaOH solution until the pH reached 11. Carboxylate groups were quantified from the titration curves according to the formula

$$\text{Oxidation}\;\text{degree}=\frac{\text{NaOH}\;\text{consumed}\;\left(\text{mL}\right)\;x\;\text{NaOH}\;\text{molarity}}{\text{sample}\;\text{mass}}$$

where tangent lines were drawn in Tiamo software (Metohm Nordic AB, Bromma, Sweden) to calculate the NaOH volume consumed. The titrations were performed as triplicates and the calculated average was reported with standard deviations as mmol*/*g of the dry material. The average nanofiber width from height images of the nanofiber fractions of TEMPO-oxidized nanocellulose was calculated using atomic force microscopy (AFM) images as 1.9 ± 0.9 nm. The material characteristics of the raw material, TEMPO-oxidized fibers and TEMPO-oxidized nanocellulose are described in previous study [49]. TC dressings were prepared following the method described [49] with a modification in filtration setup, where TEMPO-oxidized nanocellulose suspension (0.2 wt%) was magnetically stirred at RT for 1 h and degassed for 12 h under vacuum. The suspension was poured on filter membrane (Whatman grade 52, pore size 7 mm, ⌀ 90 mm) located in a sintered glass funnel connected to a vacuum pump VCP80 (VWR International AB, Stockholm, Sweden) at RT and the networks (TC) were obtained by the end of 24 h filtration in dry condition. TC dressings were rehydrated in milliQ water before use. Both BC and TC dressings were cut in 6 or 12 mm disks using a biopsy punch for subsequent use.

**Scanning Electron Microscopy (SEM):** The samples were sectioned with a surgical blade prior to freeze-drying. After transferring onto a carbon tape, the samples were sputtered with platinum for 10 s. Images were taken with a Zeiss Sigma 300 (Carl Zeiss, Germany) using 3–5 kV acceleration voltage.

**Fluorescence labelling of PLNC8 αβ:** The peptides L-PLNC8 α and L-PLNC8 β were labelled with the fluorophores Sulfo-Cyanine3 NHS ester and Sulfo-Cyanine5 NHS ester (Lumiprobe Corporation, Cockeysville, United States) respectively to allow for quantification via fluorescence spectroscopy. The peptides L-PLNC8 α and L-PLNC8 β (1 eq) were reacted with the fluorophores (1 eq) in sodium carbonate buffer (pH 8, 1 mM and 10 mM, respectively) for 1 h in ambient temperature according to manufacturer protocol. Then, unreacted fluorophore was quenched by reaction with Tris buffer (1M, 10 μL) for 30 min. Purification of conjugated peptides from unreacted fluorophore was performed indifferently via gel filtration chromatography (PD MiniTrap G-10 columns, Cytiva, Uppsala, Sweden) or via dialysis (MWCO 0.5-1kD dialysis dressing, Spectra/Por, Spectrum Laboratory Products Inc., United States).

**Peptide aggregation determination by self-quenching and FRET:** Peptides PLNC8 α and PLNC8 β were labelled with the fluorophores Sulfo-Cyanine3 NHS ester and Sulfo-Cyanine5 NHS ester (Lumiprobe GmbH, Hannover, Germany), respectively. Two tests were carried out. In a first instance, peptide PLNC8 α-Cy3 and PLNC8 α were mixed in ratio 1:69 and 1:349 (to a final concentration of 50 μM and 250 μM respectively) in milliQ water. Labelled and unlabeled peptides (PLNC8 α/PLNC8 α-Cy3 and PLNC8 β/PLNC8 β-Cy5) were mixed at a ratio of 1:69 and 1:349 to a final concentration of 50 μM and 250 μM respectively (PLNC8 α/PLNC8 β = 1:1) in milliQ water. The two solutions were incubated for 1 h, then fluorophore Cy3 was excited at 520 nm with a Fluoromax-4 spectrophotometer (Horiba Jobin Yvon Inc., United States) and emission spectra were collected at λ = 540–700 nm.

**Determination of CMC by dynamic light scattering (DLS):** Peptide stock solutions (1 mM, prepared in milliQ water) were diluted to the intended concentration (0.001–100 μM) in a cylindrical glass cuvette in milliQ water, saline solution (0.9 % NaCl), PBS+10 % FBS or LB broth filtered using a 0.22 μm filter to remove dust particles. The samples were equilibrated at a 22 °C in a water bath for at least 10 min, then sonicated for 1 min and incubated for an additional 5 min prior to being analyzed using an ALV/CGS-8F platform-based goniometer system equipped with a 632.8 mm HeNe laser (ALV-GmbH, Langen, Germany). The experiments were carried out at 22 °C using a water circulator. Scattered light was collected at a 90° angle and 10 runs of 30 s were performed and manually averaged. Data were analyzed with an ALV-Correlator software (3.0, ALV-GmbH, Langen, Germany).

**AMP loading in cellulose dressings:** BC and TC dressings were cut into 6 mm disks with a biopsy punch (Sodexo, France) and rinsed in milliQ water for at least 30 min. Labelled peptides were combined with their unlabeled counterpart in ratio of 1:70; the peptides L-PLNC8 α and L-PLNC8 β were then combined in a molar ratio 1:1 in milliQ water at concentrations 25, 50 and 250 μM (pH 5). PLNC8 αβ loading was achieved by immersion of each three 6 mm dressings in 1 mL of peptide solution. The loading was measured after 1 h, 2 h, 4 h, 8 h and 24 h of incubation at room temperature on gentle shaking (KS 260 basic orbital shaker, IKA, Staufen, Germany). Peptide loading was derived from labelled peptide depletion in the incubation solution, measured via absorbance spectroscopy (Tecan Infinite M1000 Pro, Tecan Austria GmbH, Grödig/Salzburg, Austria).

**PLNC8 αβ release from BC/TC dressings:** Peptide loaded BC/TC/BC-MSN dressings were incubated in 250 μL of PBS supplemented with 10 % fetal bovine serum (FBS) for 8 h at 37 °C. Peptide release was measured every 1 h, 2 h, 4 h, 6 h and 8 h by measuring the absorbance of the incubation solution with a Tecan Infinite M1000 Pro plate reader (Tecan Austria GmbH, Grödig/Salzburg, Austria).

**Synthesis of MSNs:** MSN were synthesized using the protocol of Björk et al. [66] Briefly, 2.4 g of Pluronic P123 (PEO_20_PPO_70_PEO_20_, M_n_ ∼5800) and 28 mg of ammonium fluoride was dissolved in 80 mL 1.83 M HCl at 20 °C [66]. Briefly, 2.4 g of Pluronic P123 (PEO_20_PPO_70_PEO_20_, M_n_ ∼5800) and 28 mg of ammonium fluoride was dissolved in 80 mL 1.83 M HCl at 20 °C. 1 mL of heptane and 5.5 mL of tetraethyl orthosilicate were mixed and added to the micelle solution under stirring. After 4 min, the stirring was turned off and the mixture was kept in static conditions over night. The solution was then transferred to a PTFE bottle for hydrothermal treatment at 100 °C for 24 h. The material was collected by filtration, thoroughly washed with deionized water, and dried at 80 °C prior to calcination at 550 °C for 5 h.

**Transmission electron microscopy (TEM):** The MSNs were suspended in ethanol and dropwise added to a hollow carbon grid. TEM micrographs were recorded using a FEI Tecnai G2 TF 20 UT (FEI Company, Hillsboro, OR, USA) microscope operated at 200 kV.

**Self-assembly of MSNs in BC/TC:** Self-assembly of MSNs in BC and TC dressings was performed as previously described [51,62]. Briefly, hydrated BC and TC dressings were cut into 6 mm discs with the help of a biopsy punch and rinsed in milliQ water. A 5 mg/mL suspension of SBA-15 was prepared in milliQ water and sonicated extensively to ensure good dispersion. Each dressing was incubated in 0.5 mL of SBA-15 suspension in an Erlenmeyer flask for 5 days under shaking (100 min^−1^ on orbital shaker) at RT. The dressings were then rinsed twice in milliQ water prior to use.

**Solvent-casted TC-MSN dressings:** TC-composite dressings were prepared by solvent casting with a protocol from Berglund et al. [49] following minor adaptations. 0.51 wt % TC gels were combined with appropriate volumes of 10 mg/mL MSN suspension in milliQ water, to yield 200 μL gels with 0 mg/mL, 0.1 mg/mL, 1 mg/mL and 10 mg/mL MSN content. The gels were homogenized by extensive vortexing and sonication (10 min, Ultrasonic Cleaner USC-TH, VWR, Avantor, Radnor, United States). Once degassed, the gels were transferred in 6 mm Ø custom-made moulds, placed in a glass open-mouth vessel, positioned on a 37 °C hotplate and allowed to dry.

**In situ MSN synthesis in BC:** The MSNs were synthesized as above [78], but 30 s into the static conditions, the solution was poured on hydrated BC for incubation over night at 20 °C followed by hydrothermal treatment at 60 °C for 24 h. The BC dressings were washed with EtOH 99 % at 80 °C for 24 h to fully remove P123, prior to being freeze-dried for analysis. The changes in hydrothermal treatment temperature and method for P123 removal compared to the free MSN synthesis were done to not damage the BC.

**Nitrogen physisorption measurements:** Nitrogen physisorption at −196 °C was used to calculate the specific surface area of the materials. The measurements were performed on an ASAP2020 (Micromeritics). The specific surface area was calculated using the BET-method at P/P_0_ = 0.07–0.18. Prior to the measurements, the samples were degassed at 200 °C for 4 h (MSNs) or freeze dried and degassed at 110 °C for 6 h (BC and BC-MSN composites). The pore size distribution was calculated using the KJS method on the adsorption isotherm, and the total pore volume was determined at P/P0 = 0.99.

**Thermogravimetric analysis (TGA):** To determine MSN loading on cellulose dressings, thermogravimetric analysis was employed. The samples were prepared as described above and carefully washed prior to being air-dried on a Teflon support (n = 10). A STA 449C Jupiter Thermo-microbalance (Netzsch-Gerätebau GmbH, Selb, Germany) equipped with an open alumina crucible was employed. Samples were heated between 50 °C and 550 °C (10 K/min heating rate in ambient atmosphere). Results were acquired via Proteus Analysis Software (Netzsch-Gerätebau GmbH, Selb, Germany).

**Water vapor transmission rate (WVTR):** The standard SS-EN 13726-2 was employed with minor modifications to determine WVTR. BC, TC and BC-MSN dressings were equilibrated in milliQ water, then cut in 12 mm disks with a surgical blade (n ≥ 4). Glass vials were filled with milliQ water (3 mL, 37 °C). The dressings were positioned on the opening of the vials and a lid with a 64 mm^2^ orifice was used to secure the dressings in place. Paraffin film was used to seal the lid to prevent any additional moisture loss. The samples were placed in an incubator (37 °C, 32 % RH) and periodically weighted over a period of 3 days to determine mass loss. Data recorded during the first 5 h were disregarded due to the influence of dressing drying on the recorded weight loss. Data was linearly fitted and normalized against dressing area. Data analysis was performed with Prism 10.0.2 software (GraphPad, *LaJolla, US*) using a one-way ANOVA complemented with Tukey's multiple comparison test (n ≥ 4).

**Water retention rate (WRR):** BC, TC and BC-MSN dressings were equilibrated in milliQ water prior to the testing (n ≥ 5). Excess water was removed by pipette aspiration and by gently blotting on wet tissue paper. The samples were then transferred onto paraffin film and the initial weight was evaluated ($W_{w}$). The samples were stored in an open mouth vessel and allowed to dry in room temperature until complete drying. Mass loss was recorded every 30 min ($W_{i}$, instantaneous weight) for a period of 3 h. The samples were then transferred to a 60 °C incubator and allowed to dry completely. The dry weight ($W_{d}$) was then recorded. WRR was calculated with the formula:

$$WRR=\left(W_{i}-W_{d}\right)/\left(W_{w}-W_{d}\right)*100\%$$

**PLNC8 αβ loading in MSNs:** A suspension of MSN was prepared in milliQ water and sonicated to reach complete homogenization. Each peptide was mixed with its labelled counterpart at suitable ratio (PLNC8 α-Cy3: PLNC8 α and PLNC8 β-Cy5: PLNC8 β) and evaluated separately. The peptide stock solutions were added to a final volume of 500 μL (10 mg/mL MSN final concentration, 200 μM peptide final concentration). The pH was adjusted to 3, 5 and 7.4 prior to the beginning of the test. Loading was allowed to occur at RT on orbital shaker (KS 260 basic orbital shaker, IKA, Staufen, Germany) for 24. The samples were centrifuged, and the supernatant was collected, and the absorbance was measured with a microplate reader (Tecan Infinite M1000 Pro, Tecan Austria GmbH, Grödig/Salzburg, Austria) to assess peptide loading at every timepoint (0 h, 1 h, 2 h, 4 h, 8 h and 24 h). After pH optimization, MSN were suspended in milliQ water and thoroughly sonicated/vortexed. Labelled PLNC8 α-Cy3 and PLNC8 β-Cy5 were mixed in ratio 1:58 with their respective unlabeled counterpart in milliQ water. The peptides were separately loaded onto MSN (500 μL final volume, 10 mg/mL final MSN concentration, 0.2 mM, 0.4 mM and 1 mM PLNC8 x concentration, n = 3). The pH was adjusted to 5 and the solution was incubated under shaking (KS 260 basic orbital shaker, IKA, Staufen, Germany) in room temperature for 24 h. At each timepoint (0 h, 1 h, 2 h, 4 h, 8 h, 24 h) the solution was centrifuged to separate the nanoparticles, the supernatant was collected, and its absorbance was measured with a microplate reader (Tecan Infinite M1000 Pro, Tecan Austria GmbH, Grödig/Salzburg, Austria). The test was concluded after 24 h when loading plateau was reached.

**Release of PLNC8 αβ from MSNs:** To simulate release in non-exudating wounds, the peptide-loaded MSNs were incubated in PBS+10 % FBS medium at concentration of 4 mg/mL, at pH 5, 7.4 and 9 (n = 3). The samples were incubated at 37 °C under shaking (MR-1 Mini-Rocker Shaker, Biosan, Riga, Latvia) for up to 8 h. The samples were centrifuged (Universal 320R Centrifuge, Hettich, Tuttlingen, Germany), and supernatant was collected, and absorbance was measured with a microplate reader (Tecan Infinite M1000 Pro, Tecan Austria GmbH, Grödig/Salzburg, Austria) at timepoint 1 h, 2 h 4 h and 8 h to determine peptide release. To simulate exudating wounds, the release was evaluated as above, with the renewal of half the release buffer every 2 h for a total of 8 h (n = 3).

**Loading of PLNC8 α/β in BC-MSN:** 6 mm Ø BC-MSN dressings were prepared as described above. Labelled PLNC8 α-Cy3 and PLNC8 β-Cy5 were mixed with their respective unlabeled counterpart to a final concentration of 50 μM, 250 μM and 1 mM (PLNC8 α/β 1:1 in milliQ water). Each BC-MSN dressing was incubated in 333 μL, the pH was adjusted to 5 and the solution was incubated under shaking (.) in room temperature for 24 h, n = 3. The absorbance of the supernatant was measured with a microplate reader (Tecan Infinite M1000 Pro, Tecan Austria GmbH, Grödig/Salzburg, Austria) at 0 h, 1 h, 2 h, 4 h, 6 h, 8 h, 24 h to determine peptide loading.

**Release of PLNC8 α/β from BC-MSN:** Loaded BC-MSN dressings were incubated in 250 μL of PBS supplemented with 10 % FBS at pH 5, 7.4 or 9 (n = 3). The incubation was let proceed for 8 h in 37 °C incubator, under shaking (MR-1 Mini-Rocker Shaker, Biosan, Riga, Latvia). Every timepoint (1 h, 2 h 4 h and 8 h) the supernatant was collected, and the absorbance was measured with a microplate reader (Tecan Infinite M1000 Pro, Tecan Austria GmbH, Grödig/Salzburg, Austria) to determine peptide release. To simulate exudative-wound conditions, the release was evaluated as described above, while renewing half the release buffer every 8 h for a total of four times (n = 3).

**In vitro antibacterial testing of PLNC8 αβ-loaded dressings:** BC, TC, and BC-MSN dressings loaded with PLNC8 αβ at different concentrations were evaluated for their antibacterial activity by either directly inoculating *S. aureus* on top of the dressings, or by performing broth microdilution MIC tests with PLNC8 αβ released from the dressings when placed in a buffer solution. To examine the direct bacterial inhibition of the surface of the peptide loaded dressings, triplicates of 6 mm diameter pieces of either BC, TC, or BC-MSN dressings were loaded with PLNC8 αβ at various concentrations and placed on a MH-agar plate, after which 5 μL of bacterial suspension containing approximately 10^5^ CFU of *S. aureus* was directly inoculated on top of the dressings. The plates were then incubated overnight at 37 °C, and stereomicroscopy photos were taken. The photos were then manually analyzed using ImageJ software to determine the percentage of bacterial coverage on the dressings and compared to the unloaded controls and colored using GIMP software (2.10.38, GNU manipulation program). To examine the antibacterial activity of released peptides from the dressings, peptide loaded dressings were removed from the loading solution and rinsed in milliQ water, and excess fluid was removed by careful pipetting, after which the dressings were placed in separate enclosed tubes containing 250 μL of either saline solution or PBS +10 % FBS. The tubes containing the samples and release buffer were then placed at 37 °C for 24 h under static conditions. After 24 h, the dressings were discarded and the tubes containing the release buffer and leeched peptides were sonicated for 3∗15 s. A standardized MIC test (according to EUCAST standards) was then performed. Briefly, 100 μl of release sample was combined with 100 μL of LB broth containing bacteria to reach a final volume of 200 μL per well, each containing 10^5^ CFU of *S. aureus*. A serial dilution of each sample was performed, resulting in a dilution range from 1:2 to 1:32 of each sample. The 96-well plate was then incubated on a shaker at 37 C for 24 h, after which visual MIC and absorbance at OD 620 was determined. A standard of known PLNC8 αβ concentrations was performed for each MIC test. To determine the MBC, triplicates of 10 μl from each well was collected and cultured on agar plates, and then incubated at 37 °C ON, after which the colonies were counted, and MBC determined. All statistical analyses for the *in vitro* bacterial testing were determined by one-way ANOVA with Sidak's post-hoc test using GraphPad Prism v. 9.2.0.

**Cytotoxic risk assessment of PLNC8 αβ loaded dressings:** Human primary keratinocytes and fibroblasts were isolated and cultured as described above. Cells were seeded on flat-bottom 96 well plates (Falcon, Corning Inc.) at 4000 cell/well concentration. Keratinocytes (n ≥ 7). were seeded two days prior to the beginning of the experiment, whereas fibroblasts (n = 4) were seeded one day prior to the beginning of the experiment. Peptide loaded BC, TC and BC-MSN dressings (6 mm Ø) were incubated in 250 μL of PBS + 10 % FBS in 37 °C for 8 h. 10 % of the obtained eluate-rich solution was used to supplement respectively keratinocyte and fibroblast medium for a total of 150 μL/well. The cell culture was carried out with a LiveCyte 2 kinetic cytometer (Phase Focus Ltd, Sheffield, UK, 37 °C, 5 % CO2, 95 % humidity) for 72 h with hourly monitoring. Data were processed with PF Assay Analysis v.3.7.1 software (Phase Focus Ltd, Sheffield, UK) and analyzed with Prism 8.0 (Graphpad, LaJolla, US). Cell count was normalized to starting time and was displayed as mean and standard deviation. Statistical analysis was carried on using a two-way ANOVA complemented with Tukey's multiple comparison test. Cell migration data were displayed as mean and standard deviation and statistical analysis was carried on using a one-way ANOVA complemented with Dunnett's multiple comparison test.

## CRediT authorship contribution statement

**Elisa Zattarin:** Writing – review & editing, Writing – original draft, Methodology, Investigation, Formal analysis, Conceptualization. **Zeljana Sotra:** Writing – review & editing, Writing – original draft, Methodology, Investigation, Formal analysis. **Emanuel Wiman:** Writing – review & editing, Methodology, Investigation, Formal analysis. **Yagmur Bas:** Writing – review & editing, Methodology, Investigation, Formal analysis. **Jonathan Rakar:** Writing – review & editing, Methodology, Investigation, Formal analysis. **Linn Berglund:** Writing – review & editing, Methodology, Investigation, Formal analysis. **Annika Starkenberg:** Writing – review & editing, Methodology, Investigation. **Emma M. Björk:** Writing – review & editing, Supervision, Methodology, Investigation, Formal analysis. **Hazem Khalaf:** Writing – review & editing, Supervision, Methodology, Investigation, Formal analysis. **Kristiina Oksman:** Writing – review & editing, Methodology, Funding acquisition, Formal analysis, Conceptualization. **Torbjörn Bengtsson:** Writing – review & editing, Writing – original draft, Supervision, Funding acquisition, Formal analysis, Conceptualization. **Johan P.E. Junker:** Writing – review & editing, Writing – original draft, Supervision, Methodology, Investigation, Funding acquisition, Formal analysis, Conceptualization. **Daniel Aili:** Writing – review & editing, Writing – original draft, Supervision, Resources, Methodology, Funding acquisition, Formal analysis, Conceptualization.

## Declaration of competing interest

The authors declare that they have no known competing financial interests or personal relationships that could have appeared to influence the work reported in this paper.

## Data Availability

Data will be made available on request.

## References

[1] Bowler P.G. (2002). Ann. Med..

[2] Klein T.M., Andrees V., Kirsten N., Protz K., Augustin M., Blome C. (2021). Int. Wound J..

[3] Kopecki Z. (2021). Biosci. Rep..

[4] Zheng L., Li S., Luo J., Wang X. (2020). Front. Bioeng. Biotechnol..

[5] Posnett J., Gottrup F., Lundgren H., Saal G. (2009).

[6] Hopmans T.E.M., Blok H.E.M., Troelstra A., Bonten M.J.M. (2007). Infect. Control Hosp. Epidemiol..

[7] Saghazadeh S., Rinoldi C., Schot M., Kashaf S.S., Sharifi F., Jalilian E., Nuutila K., Giatsidis G., Mostafalu P., Derakhshandeh H., Yue K., Swieszkowski W., Memic A., Tamayol A., Khademhosseini A. (2018). Adv. Drug Deliv. Rev..

[8] Pfalzgraff A., Brandenburg K., Weindl G. (2018). Front. Pharmacol..

[9] Patrulea V., Borchard G., Jordan O. (2020). Pharmaceutics.

[10] De Oliveira D.M.P., Forde B.M., Kidd T.J., Harris P.N.A., Schembri M.A., Beatson S.A., Paterson D.L., Walker M.J. (2020). Clin. Microbiol. Rev..

[11] Mangoni M.L., McDermott A.M., Zasloff M. (2016). Exp. Dermatol..

[12] Kumar P., Kizhakkedathu J.N., Straus S.K. (2018). Biomolecules.

[13] Mahlapuu M., Håkansson J., Ringstad L., Björn C. (2016). Front. Cell. Infect. Microbiol..

[14] Atefyekta S., Blomstrand E., Rajasekharan A.K., Svensson S., Trobos M., Hong J., Webster T.J., Thomsen P., Andersson M. (2021). ACS Biomater. Sci. Eng..

[15] Lozeau L.D., Grosha J., Kole D., Prifti F., Dominko T., Camesano T.A., Rolle M.W. (2017). Acta Biomater..

[16] Cassin M.E., Ford A.J., Orbach S.M., Saverot S.E., Rajagopalan P. (2016). Acta Biomater..

[17] Yuan J., Zhang D., He X., Ni Y., Che L., Wu J., Wu B., Wang Y., Wang S., Sha D., Zheng S.Y., Yang J. (2021). Int. J. Biol. Macromol..

[18] Yasir M., Dutta D., Hossain K.R., Chen R., Ho K.K.K., Kuppusamy R., Clarke R.J., Kumar N., Willcox M.D.P. (2020). Front. Microbiol..

[19] Thapa R.K., Diep D.B., Tønnesen H.H. (2020). Acta Biomater..

[20] Travkova O.G., Moehwald H., Brezesinski G. (2017). Adv. Colloid Interface Sci..

[21] Sulaeva I., Henniges U., Rosenau T., Potthast A. (2015). Biotechnol. Adv..

[22] Cattelaens J., Turco L., Berclaz L.M., Huelsse B., Hitzl W., Vollkommer T., Bodenschatz K.J. (2020). Life.

[23] Vindenes H., Bjerknes R. (1995). Burns.

[24] Jančič U., Trček J., Verestiuc L., Vukomanović M., Gorgieva S. (2024). Int. J. Biol. Macromol..

[25] Fürsatz M., Skog M., Sivlér P., Palm E., Aronsson C., Skallberg A., Greczynski G., Khalaf H., Bengtsson T., Aili D. (2018). Biomed. Mater..

[26] van Zyl E.M., Coburn J.M. (2024). Int. J. Mol. Sci..

[27] Weishaupt R., Zünd J.N., Heuberger L., Zuber F., Faccio G., Robotti F., Ferrari A., Fortunato G., Ren Q., Maniura-Weber K., Guex A.G. (2020). Adv. Healthcare Mater..

[28] Kim G., Yoon H., Park Y. (2010). Appl. Phys. Mater. Sci. Process.

[29] Afshar A., Yuca E., Wisdom C., Alenezi H., Ahmed J., Tamerler C., Edirisinghe M. (2021). Med. Devices Sensors.

[30] Heunis T., Bshena O., Klumperman B., Dicks L. (2011). Int. J. Mol. Sci..

[31] Heunis T.D.J., Smith C., Dicks L.M.T. (2013). Antimicrob. Agents Chemother..

[32] Ahire J.J., Dicks L.M.T. (2015). Probiotics Antimicrob. Proteins.

[33] Laverty G., Gorman S.P., Gilmore B.F. (2012). J. Biomed. Mater. Res., Part A.

[34] Li X., Fan R., Tong A., Yang M., Deng J., Zhou L., Zhang X., Guo G. (2015). Int. J. Pharm..

[35] Obuobi S., Tay H.K.L., Tram N.D.T., Selvarajan V., Khara J.S., Wang Y., Ee P.L.R. (2019). J. Contr. Release.

[36] McCarty S.M., Percival S.L. (2013). Adv. Wound Care.

[37] Maldonado A., Ruiz-Barba J.L., Jiménez-Díaz R. (2003). Appl. Environ. Microbiol..

[38] Bengtsson T., Selegård R., Musa A., Hultenby K., Utterström J., Sivlér P., Skog M., Nayeri F., Hellmark B., Söderquist B., Aili D., Khalaf H. (2020). Sci. Rep..

[39] Khalaf H., Nakka S.S., Sandén C., Svärd A., Hultenby K., Scherbak N., Aili D., Bengtsson T. (2016). BMC Microbiol..

[40] Hong S.Y., Oh J.E., Lee K.H. (1999). Biochem. Pharmacol..

[41] Sivlér T., Sivlér P., Skog M., Conti L., Aili D. (2018). Adv. Skin Wound Care.

[42] Aboelnaga A., Elmasry M., Adly O.A., Elbadawy M.A., Abbas A.H., Abdelrahman I., Salah O., Steinvall I. (2018). Burns.

[43] delli Santi G., Borgognone A. (2019). Burn. Open.

[44] Karlsson M., Olofsson P., Steinvall I., Sjöberg F., Thorfinn J., Elmasry M. (2019). Adv. Wound Care.

[45] Qureshi M.A., Lalwani P., Asad I., Khan-Assad N., Mohamedali S., Otour B., Chaudhary A., Mendonca D. (2021). Burn. Open.

[46] Shanks L.A., Cronshaw A., Alexander K.S., Davies J.A., O'Boyle C.P. (2020). Scars, burn. Health.

[47] Khamise A., Lapid H., Mishra A., Murray A.M. (2024). J. Plast. Reconstr. Aesthetic Surg..

[48] Tomé L.C., Brandão L., Mendes A.M., Silvestre A.J.D., Neto C.P., Gandini A., Freire C.S.R., Marrucho I.M. (2010). Cellulose.

[49] Berglund L., Squinca P., Baş Y., Zattarin E., Aili D., Rakar J., Junker J., Starkenberg A., Diamanti M., Sivlér P., Skog M., Oksman K. (2023). Biomacromolecules.

[50] Portela R., Leal C.R., Almeida P.L., Sobral R.G. (2019). Microb. Biotechnol..

[51] Eskilson O., Zattarin E., Berglund L., Oksman K., Hanna K., Rakar J., Sivlér P., Skog M., Rinklake I., Shamasha R., Sotra Z., Starkenberg A., Odén M., Wiman E., Khalaf H., Bengtsson T., Junker J.P.E., Selegård R., Björk E.M., Aili D. (2023). Mater. Today Bio.

[52] James G.A., Swogger E., Wolcott R., deLancey Pulcini E., Secor P., Sestrich J., Costerton J.W., Stewart P.S. (2008). Wound Repair Regen..

[53] Seaton M., Hocking A., Gibran N.S. (2015). ILAR J..

[54] Grada A., Mervis J., Falanga V. (2018). J. Invest. Dermatol..

[55] Taheri-Araghi S. (2024). Front. Microbiol..

[56] Mhlongo J.T., Waddad A.Y., Albericio F., de la Torre B.G. (2023). Adv. Sci..

[57] Lee C.C., Tsai C.H., Chen C.H., Yeh Y.C., Chung W.H., Chen C.B. (2023). Front. Immunol..

[58] Mony M.P., Harmon K.A., Hess R., Dorafshar A.H., Shafikhani S.H. (2023). Cells.

[59] Velnar T., Bailey T., Smrkolj V. (2009). J. Int. Med. Res..

[60] Landén N.X., Li D., Ståhle M. (2016). Cell. Mol. Life Sci..

[61] Lahiri D., Nag M., Dutta B., Dey A., Sarkar T., Pati S., Edinur H.A., Kari Z.A., Noor N.H.M., Ray R.R. (2021). Int. J. Mol. Sci..

[62] Eskilson O., Lindström S.B., Sepulveda B., Shahjamali M.M., Güell-Grau P., Sivlér P., Skog M., Aronsson C., Björk E.M., Nyberg N., Khalaf H., Bengtsson T., James J., Ericson M.B., Martinsson E., Selegård R., Aili D. (2020). Adv. Funct. Mater..

[63] Pogorelova N., Rogachev E., Digel I., Chernigova S., Nardin D. (2020). Materials.

[64] Baş Y., Berglund L., Niittylä T., Zattarin E., Aili D., Sotra Z., Rinklake I., Junker J., Rakar J., Oksman K. (2023). Biomacromolecules.

[65] Queen D., Evans J.H., Gaylor J.D.S., Courtney J.M., Reid W.H. (1987). Biomaterials.

[66] Björk E.M., Söderlind F., Odén M. (2013). Langmuir.

[67] Lérida-Viso A., Estepa-Fernández A., García-Fernández A., Martí-Centelles V., Martínez-Máñez R. (2023). Adv. Drug Deliv. Rev..

[68] Croissant J.G., Fatieiev Y., Khashab N.M. (2017). Adv. Mater..

[69] Queen D., Gaylor J.D.S., Evans J.H., Courtney J.M., Reid W.H. (1987). Biomaterials.

[70] Liang Z., Lai P., Zhang J., Lai Q., He L. (2023). Int. Wound J..

[71] Metcalf D.G., Haalboom M., Bowler P.G., Gamerith C., Sigl E., Heinzle A., Burnet M.W.M. (2019). Wound Med.

[72] Dargaville T.R., Farrugia B.L., Broadbent J.A., Pace S., Upton Z., Voelcker N.H. (2013). Biosens. Bioelectron..

[73] Jones E.M., Cochrane C.A., Percival S.L. (2014). Adv. Wound Care.

[74] Haidari H., Melguizo-Rodríguez L., Cowin A.J., Kopecki Z. (2023). Am. J. Physiol. Cell Physiol..

[75] Moya S.E., Hernández R.R., Angelomé P.C. (2024). Adv. NanoBiomed Res..

[76] Rheinwald J.G., Green H. (1975). Cell.

[77] Jonasson S., Bünder A., Niittylä T., Oksman K. (2020). Cellulose.

[78] Björk E.M., Söderlind F., Odén M. (2014). J. Colloid Interface Sci..

