# Supplementary material for: Controlled release of antimicrobial peptides from nanocellulose wound dressings for treatment of wound infections
Source: Mater Today Bio. 2025 Apr 10;32:101756. doi: 10.1016/j.mtbio.2025.101756 (PMC12032947; doi:10.1016/j.mtbio.2025.101756)
Supplement: Multimedia component 1 [file mmc1.pdf]

Supporting Information

Controlled Release of Antimicrobial Peptides from Nanocellulose Wound Dressings for Treatment of Wound Infections

Elisa Zattarin,[1] Zeljana Sotra,[2] Emanuel Wiman,[3] Yağmur Baş,[4] Jonathan Rakar,[2] Linn Berglund,[4] Annika Starkenberg,[2] Emma M. Björk,[5] Hazem Khalaf,[3] Kristiina Oksman,[4] Torbjörn Bengtsson[3] Johan P.E. Junker,[2,] Daniel Aili[1;*]*

[1] Laboratory of Molecular Materials, Division of Biophysics and Bioengineering, Department of Physics, Chemistry and Biology, Linköping University, SE-581 83 Linköping, Sweden.

[2] Center for Disaster Medicine and Traumatology, Department of Biomedical and Clinical Sciences, Linköping University, SE-581 85 Linköping, Sweden.

[3] Department of Microbiology, Immunology and Reproductive Science, School of Medical Sciences, Örebro University, SE-70362 Örebro, Sweden.

[4] Division of Materials Science, Department of Engineering Sciences and Mathematics, Luleå University of Technology, SE-971 87 Luleå, Sweden.

[5] Division of Nanostructured Materials, Department of Physics, Chemistry and Biology (IFM), Linköping University, SE-58183 Linköping, Sweden.

*Corresponding authors: daniel.aili@liu.se, johan.junker@liu.se

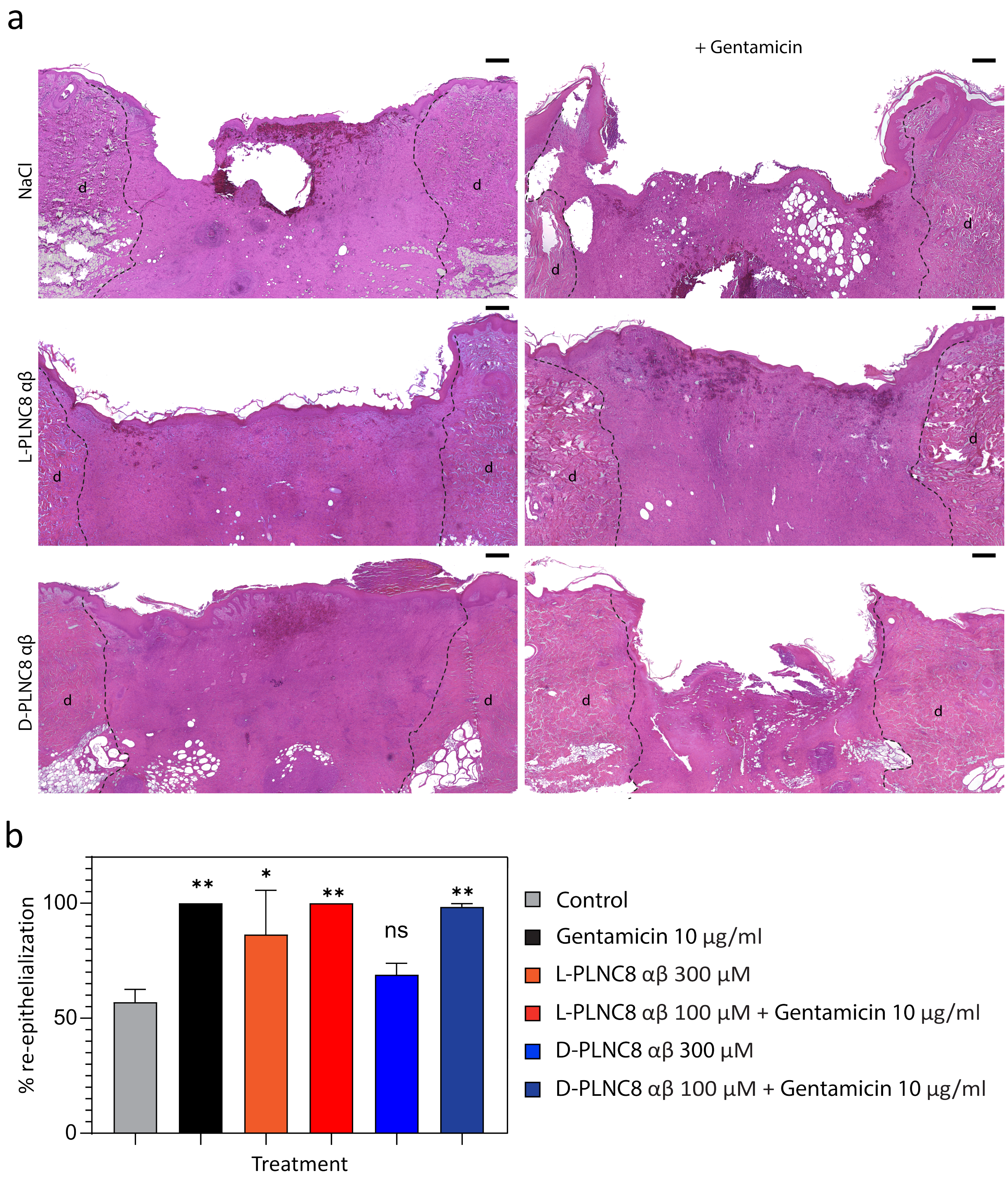

Figure S1. Histology. a) Biopsies of partial-thickness wounds stained with Hematoxylin-Eosin (HE) eight days after start of treatment. The dashed lines delineate original wound borders, and adjacent healthy dermis is indicated by (d). Scale bar: 500 µm. b) Re-epithelialization of wounds eight days after start of treatment. Treatment significantly accelerated re-epithelialization compared to control, with the exception of D-PLNC8 αβ.

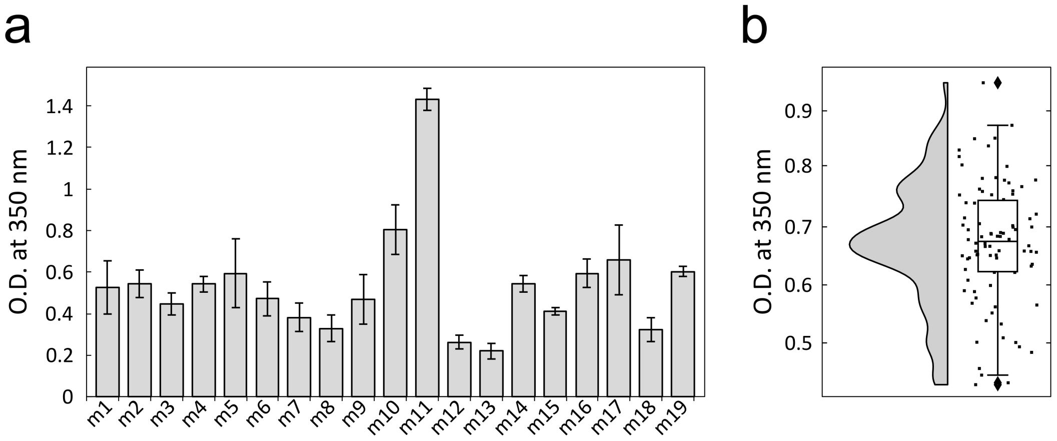

Figure S2. BC thickness. Optical density measurements of bacterial cellulose dressings at 350 nm, illustrating the variation in thickness of a) 19 different BC dressings (m1-m19), b) and in different position of one single BC dressing.

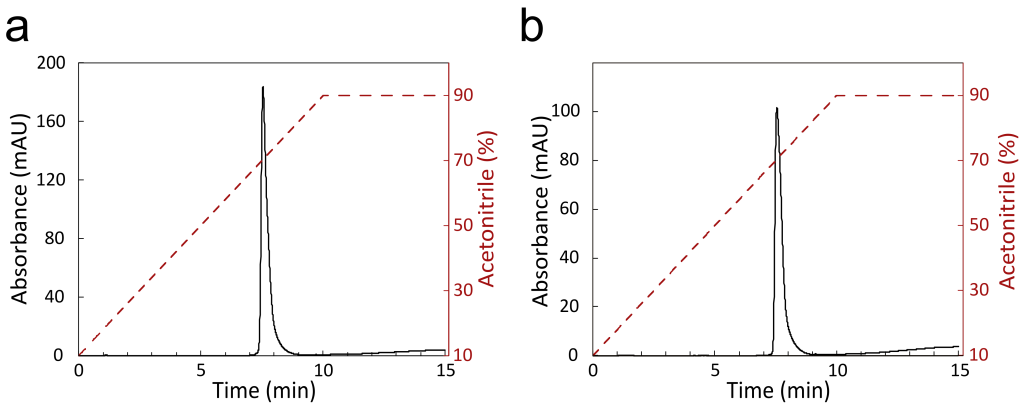

Figure S3. Peptide purity. HPLC traces of a) *L*-PLNC8 a and b) *L*-PLNC8 b. Chromatograms were acquired using a XBridge BEH Phenyl Hexyl column (Waters Corporation), at 18 ml/min flow rate, using an aqueous gradient of acetonitrile (10%–90%) containing 0.1% TFA over 10 min, followed by a linear flow of 90% acetonitrile for 5 min (secondary vertical axis, dotted line).

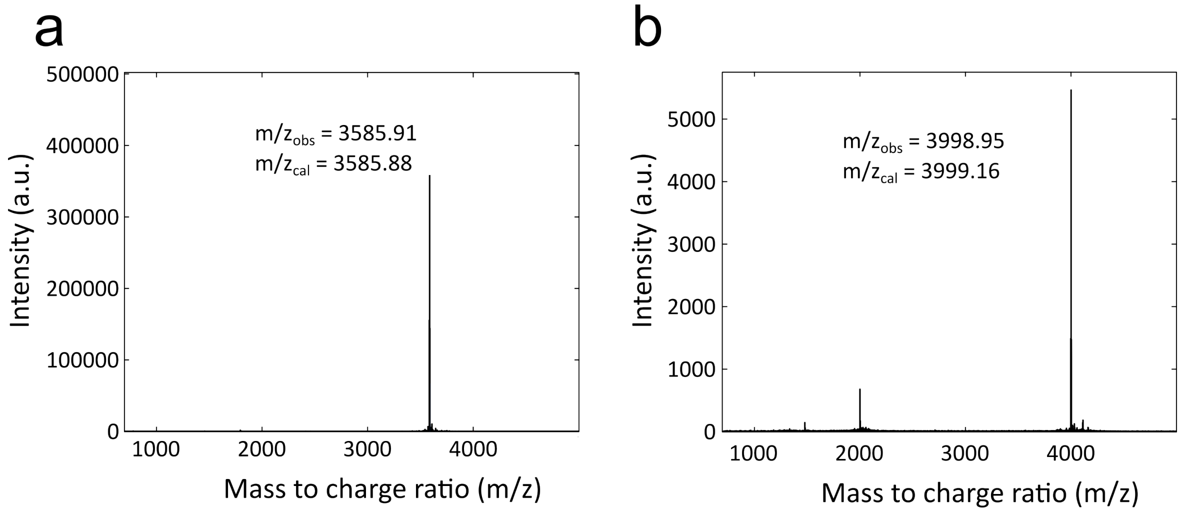

Figure S4. Peptide identity. MALDI-ToF MS of a) L-PLNC8 α and b) L-PLNC8 β.

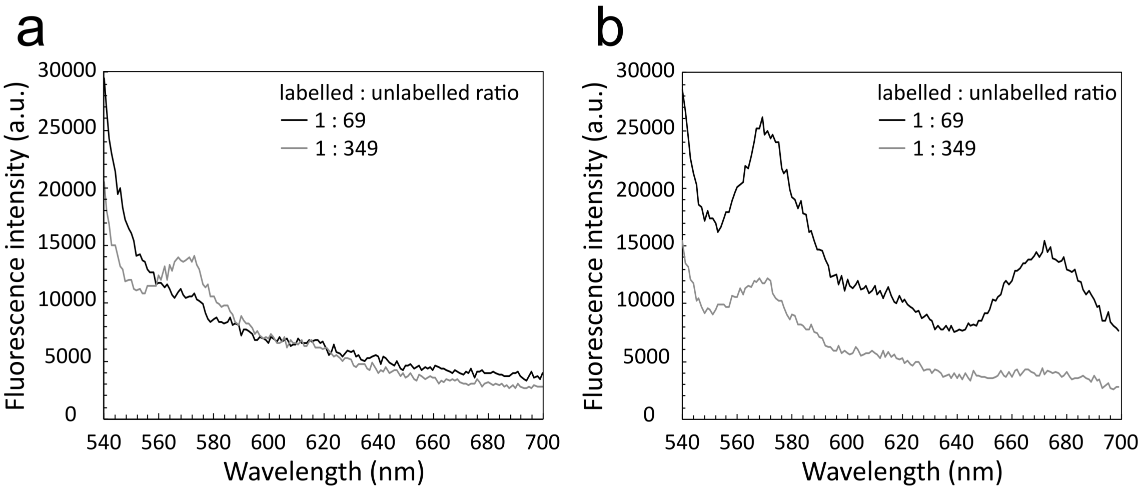

Figure S5. Self-quenching and FRET of CY3/5 labelled peptides. Emission spectra of a) PLNC8 α-Cy3 (λ_{ex}=520 nm, PLNC8 α-cy3:PLNC8 α = 1:69 and 1:349) after 1h incubation, and b) PLNC8 α-Cy3 (λ_{ex}=520 nm, PLNC8 α: PLNC8 β = 1:1, with PLNC8 α-Cy3:PLNC8 α and PLNC8 β-Cy5 : PLNC8 β = 1:69 and 1:349) after 1 h incubation.

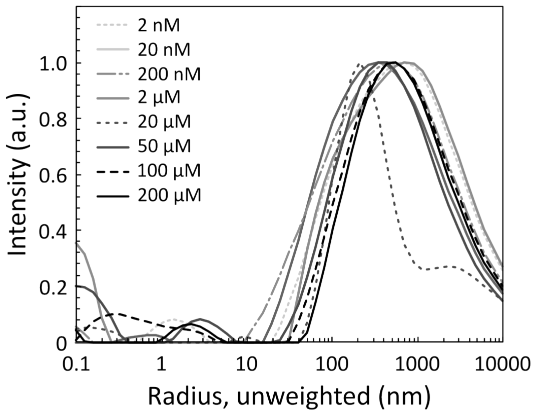

Figure S6. **Hydrodynamic radius of PLNC8 αβ micelles**. Hydrodynamic radius distribution of PLNC8 αβ micelles evaluated by dynamic light scattering (DLS). PLNC8 α/β (1:1) was prepared in milliQ water, at total concentration 2 nM - 200 μM.

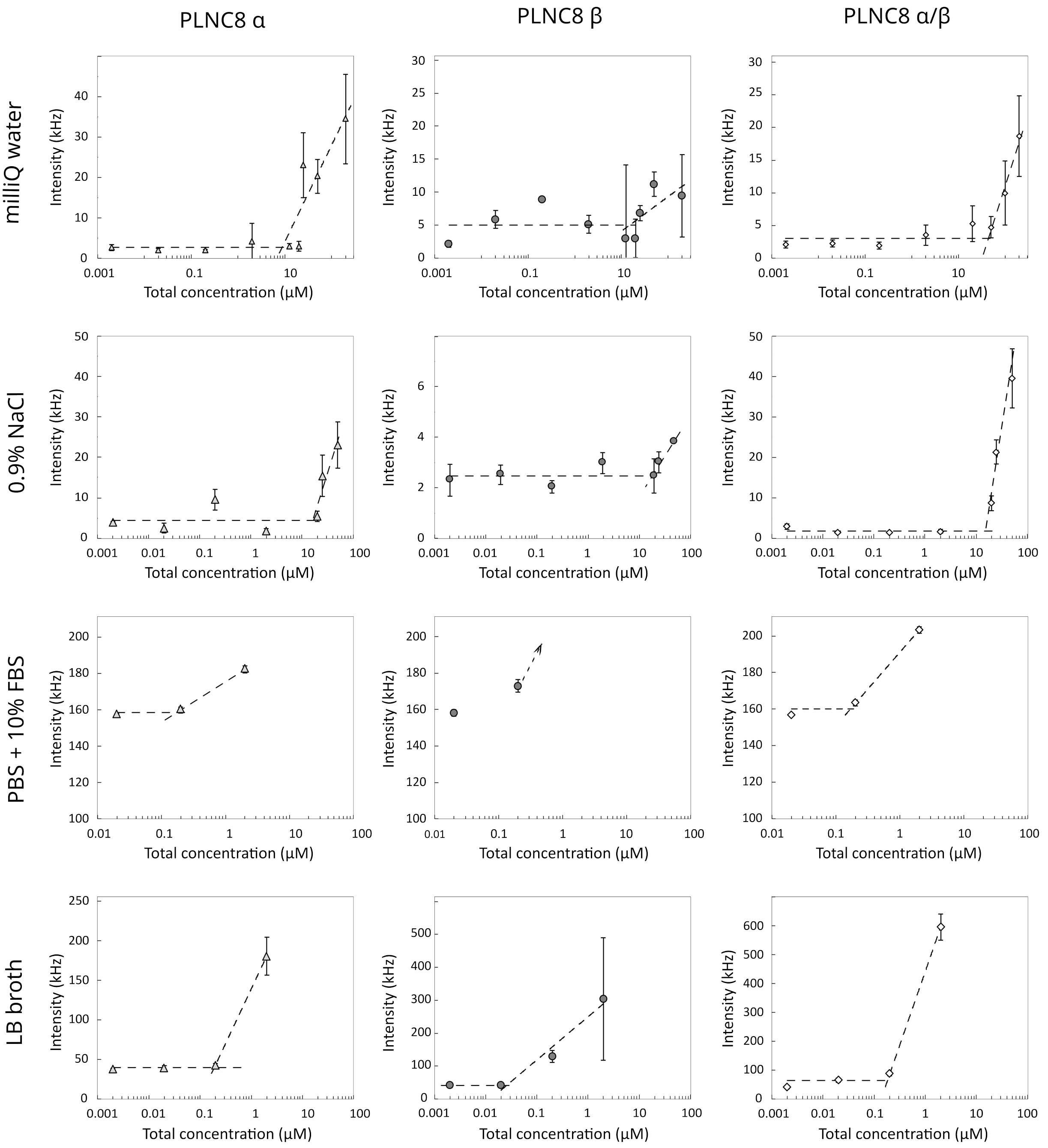

Figure S7. Scattering intensity of PLNC8 αβ. Changes in scattering intensity by dynamic light scattering (DLS) of the peptides PLNC8 α, PLNC8 β and PLNC8 α/β (1:1) as a function of peptide concentration in the solvents: milliQ water, saline, PBS + 10% FBS and LB broth.

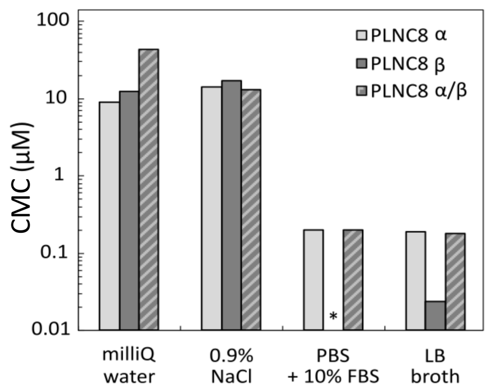

Figure S8. CMC of PLNC8 αβ. CMC of the peptides PLNC8 α, PLNC8 β and PLNC8 α/β (1:1) in milliQ, saline, PBS + 10% FBS, and LB borth, as determined by DLS analysis in Figure S5 (* ND).

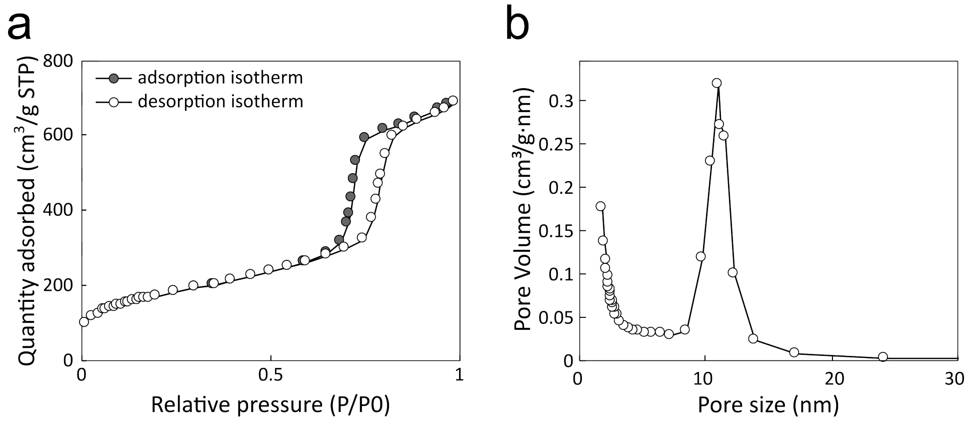

Figure S9. MSN characterization. a) Nitrogen physisorption isotherms and b) pore size distribution of MSN SBA-15

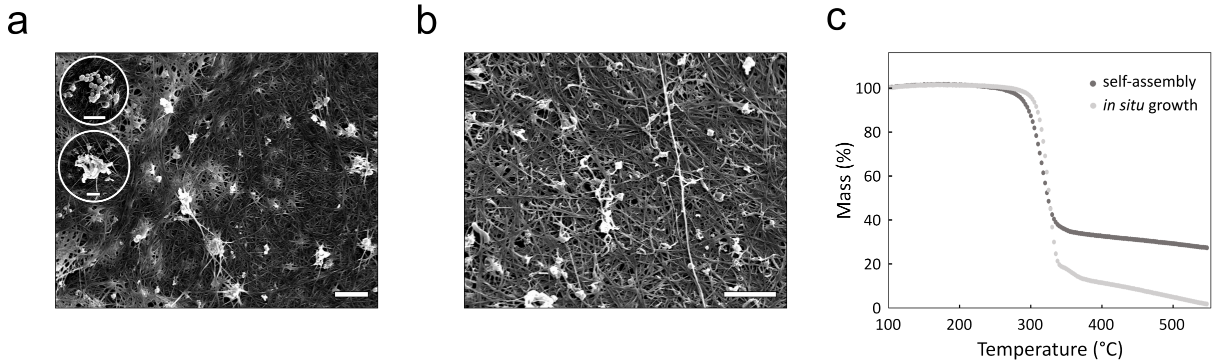

Figure S10. In situ MSN growth on BC dressings. a-b) SEM micrographs of a) BC-MSN containing P123 (unwashed). Inset: variation of MSN morphology within the dressing, and b) BC-MSN to which P123 was removed (washed), scale bar: 1 µm. c) TGA of BC dressings functionalized with MSN by self assembly (incubation of 1 BC dressing in 0.5 mL of 5 mg/mL MSN for 5 days) and in situ growth.

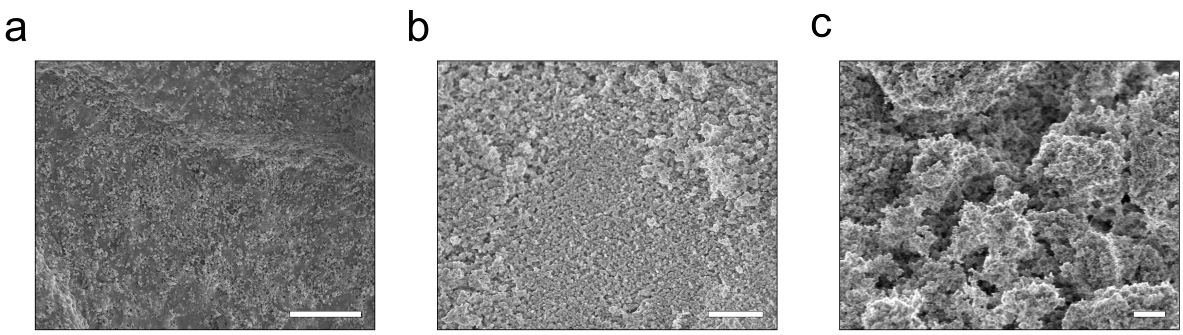

Figure S11. MSN self-assembly in BC dressings. SEM micrographs of BC dressings incubated in a) 5 mg/mL MSN for 5 days, b) 5 mg/mL MSN for 14 days and c) 10 mg/mL MSN for 14 days (scale bar 10 µm).

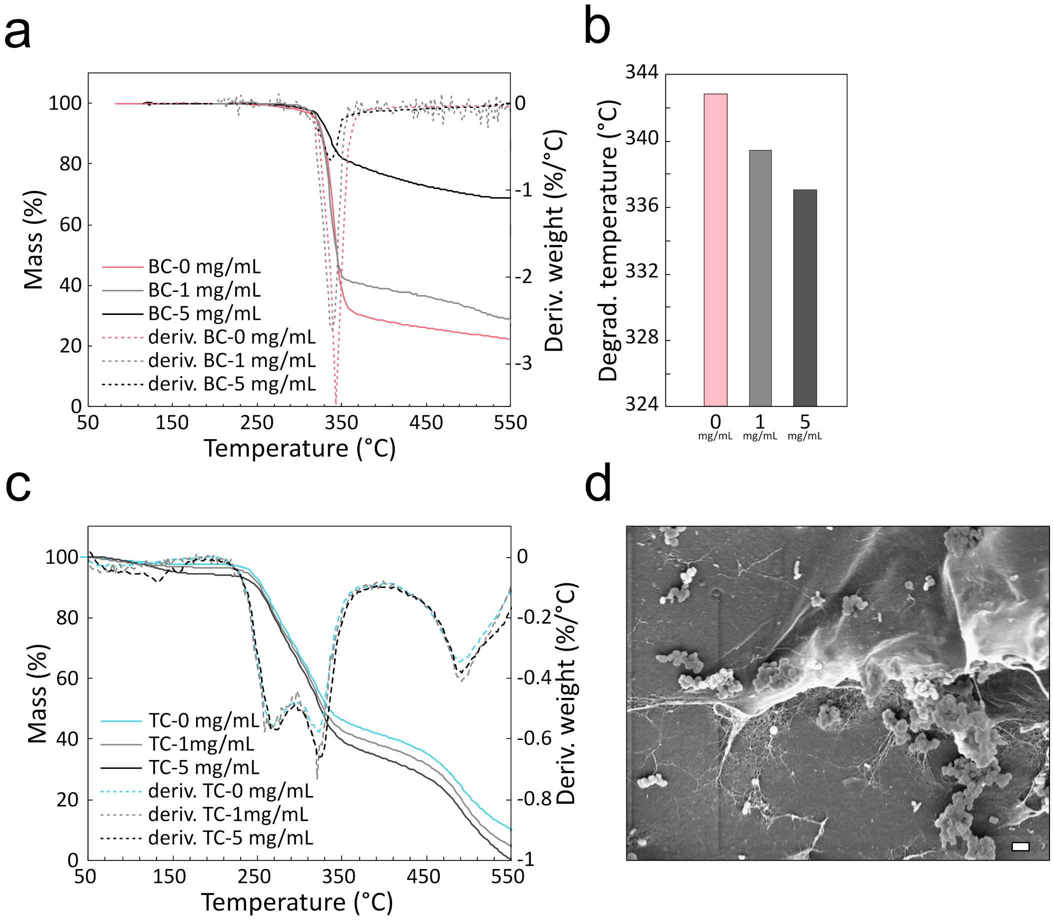

Figure S12. MSN self-assembly on BC and TC dressings. Thermogravimetric analysis of a) BC-MSN obtained by incubation of BC dressings in MSN suspension (0 mg/mL, 1 mg/mL and 5 mg/mL MSN) for 5 days, n=10. b) Degradation temperature of BC-MSN composites. c) Thermogravimetric analysis of TC-MSN obtained by incubation of TC dressings in MSN suspension (0 mg/mL, 1 mg/mL and 5 mg/mL MSN) for 5 days, n=10. d) SEM micrographs of TC hydrogels incubated in 10 mg/mL MSN for 1 day. Scale bar 1 µm.

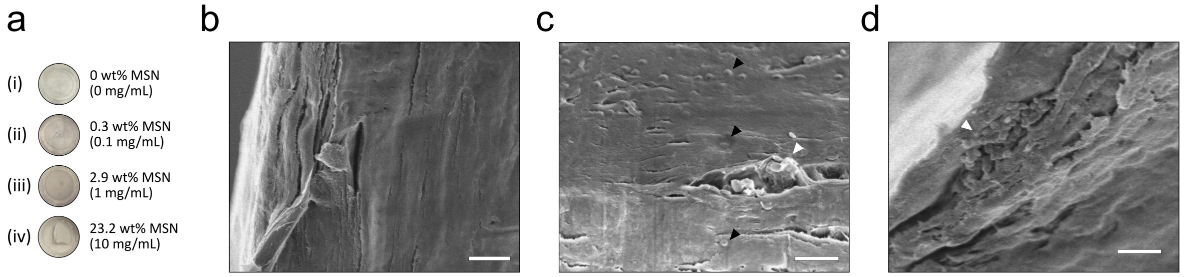

Figure S13. Solvent-casted TC-MSN dressings. a) Photographs of composite TC-MSN dressings (grammage = 20 g/m^2) prepared bottom-up via solvent casting from 0.51 wt% TC hydrogel and i) 0 mg/mL, ii) 0.1 mg/mL, iii) 1 mg/mL and iv) 10 mg/mL MSN suspension, resulting in 0, 0.3, 2.9 md 23.2 wt% MSN respectively. b-d) SEM micrographs of b) TC-MSN-0wt%, c) TC-MSN-0.3 wt% and d) TC-MSN-2.9 wt% dressings. Black arrows indicate single particles, white arrows indicate aggregates. Scale bar: 2 µm.

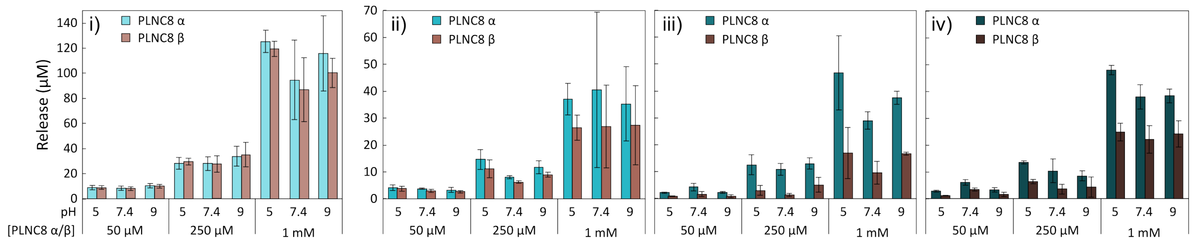

Figure S14. Peptide release from BC-MSN dressings, simulating high exudate wound conditions. i) Release of peptide PLNC8 αβ from BC-MSN wound dressing in PBS+10% FBS, pH 5, 7.4 and 9 (n=3). ii) second, iii) third and iv) fourth release (in 250 µL buffer at the corresponding pH, n=3).
